# Efficacy and durability of immediate versus delayed single-dose HPV vaccination for persistent infection among young women in Kenya: a randomized, blinded, cross-over clinical trial

**DOI:** 10.1038/s41467-026-72654-8

**Published:** 2026-05-11

**Authors:** Ruanne V. Barnabas, Elizabeth R. Brown, Elizabeth A. Bukusi, Betty Njoroge, Rachel L. Winer, Denise A. Galloway, Imeldah N. Wakhungu, Charlene Biwott, Syovata Kimanthi, Kate B. Heller, Meighan Krows, Susan Morrison, Elena A. Rechkina, Stephen L. Cherne, R. Scott McClelland, Nelly R. Mugo, Maricianah A. Onono

**Affiliations:** 1 Division of Infectious Diseases, Massachusetts General Hospital, Boston, MA USA; 2 Department of Medicine, Harvard Medical School, Boston, MA USA; 3 Department of Epidemiology, T. H. Chan Harvard School of Public Health, Boston, MA USA; 4 Ragon Institute of Massachusetts Brigham, Cambridge, MA USA; 5 Vaccine and Infectious Disease Division, Fred Hutchinson Cancer Center, Seattle, WA USA; 6 Public Health Sciences Division, Fred Hutchinson Cancer Center, Seattle, WA USA; 7 Department of Biostatistics, University of Washington, Seattle, WA USA; 8 Department of Global Health, University of Washington, Seattle, WA USA; 9 Department of Obstetrics and Gynecology, University of Washington, Seattle, WA USA; 10 Center for Microbiology Research, Kenya Medical Research Institute, Kisumu, Kenya; 11 Center for Clinical Research, Kenya Medical Research Institute, Nairobi, Kenya; 12 Department of Epidemiology, University of Washington, Seattle, WA USA; 13 Human Biology Division, Fred Hutchinson Cancer Center, Seattle, WA USA; 14 Department of Pathology, University of Washington, Seattle, WA USA; 15 Division of Allergy and Infectious Diseases, University of Washington, Seattle, WA USA

**Keywords:** Viral infection, Cancer prevention, Translational research, Vaccines

## Abstract

Evidence is needed for single-dose human papillomavirus (HPV) vaccine efficacy (VE) durability to support vaccination guidelines. In this randomized crossover trial (NCT03675256), healthy young women aged 15-20 years, recruited through community-based screening in Kenya, were randomly allocated to immediate nonavalent or bivalent HPV vaccination and delayed control at month 30/36 (age 17-23 years), or immediate control and delayed nonavalent HPV vaccination. Cervical swabs collected every six months were tested for HPV DNA to determine incident persistent HPV infection. The primary outcome was VE at 54 months, estimated among participants who were HPV naive at enrollment vaccination using Cox proportional hazards models with time-varying covariates for HPV vaccine status and time; negative coefficients for time since vaccination indicate durability. For incident persistent HPV 16/18 infections, 104 were detected: 93 pre-crossover and 11 post-crossover; HPV 16/18 VE was 99.3% (95% CI: 96.2,99.9%). For incident persistent HPV 16/18/31/33/45/52/58, 117 infections occurred: 103 pre-crossover and 14 post-crossover; HPV 16/18/31/33/45/52/58 VE was 98.9% (95% CI: 94.9,99.8%). Coefficients for time since vaccination were −0.0014 (95% CI: −0.0027,−0.0002) for HPV 16/18 and −0.0016 (95% CI: −0.0028,−0.0004) for HPV 16/18/31/33/45/52/58. Single-dose HPV vaccination is highly efficacious ( > 98%) and durable over 54 months in young women.

## Abstract (web summary)

Here, the authors report that a single Human papillomavirus (HPV) vaccine dose given to young women in Kenya is effective and confers protection for 4.5 years, preventing the HPV types that cause most cervical cancers.

## Introduction

Worldwide, in 2022, an estimated 662,044 new cervical cancer cases and 348,709 related deaths were reported^1^. Most countries have an incidence rate for cervical cancer that is higher than the World Health Organization’s (WHO’s) threshold for cervical cancer elimination, which is <4 cases/100,000 woman-years^2^. In Kenya, the annual age-standardized cervical cancer incidence is 32.8 cases/100,000 person-years. Some countries are close to achieving cervical cancer elimination; in Australia, cervical cancer incidence is 5.3 cases/100,000 person-years, reflecting the effectiveness of high-efficacy tools for prevention, diagnosis, and treatment^1^. The average age at diagnosis is 50 years^3^. Cervical cancer is frequently diagnosed at a late stage, has high mortality, and is often accompanied by economic and social consequences for families and communities^4–6^. Human papillomavirus (HPV) infection, the primary cause of cervical cancer^7,8^, is acquired during early adulthood. While most HPV infections clear, a few persist, and, over a decade; late-stage oncogenic HPV proteins cause unchecked cell proliferation and cancer in midlife^9^.

HPV vaccines prevent more than 90% of persistent oncogenic vaccine type-specific HPV infections, the primary cause of cervical cancer. Four HPV vaccines are licensed to be given as 2–3 intramuscular injections over 2–6 months, all targeting high-risk (oncogenic) HPV types that cause 70%–90% of cancers. The bivalent vaccines (Cervarix® and Cecolin®) prevent HPV 16/18 infection, the quadrivalent vaccine (Gardasil®) prevents HPV 16/18/6/11, including the low-risk HPV types 6 and 11 to prevent genital warts, and the nonavalent vaccine (Gardasil-9®) prevents HPV 16/18/31/33/45/52/58/6/11 infection, including five additional high-risk HPV types. The WHO Elimination Strategy aims to vaccinate 90% of girls prior to HPV exposure as the vaccine has no therapeutic properties^10^. HPV vaccine licensure studies report vaccine efficacy (VE) among persons without evidence of current or past HPV infection (i.e., HPV naïve). Few programs in southern African offer catch-up vaccination to persons age 15-years and older, in part due to limited VE data for that age group. Globally, in 2023, 27% of adolescent girls, age 9–24 years, received HPV vaccination; with coverage in low-and-middle-income countries substantially lower^11^. Vaccinating the current global cohort of women aged 9–18 years would prevent HPV-associated precancerous lesions^12^ and cervical cancer over their lifetimes^13^.

The vaccine virus-like-particle structure, which self-assembles to mimic the live virus without the replicating deoxyribonucleic acid (DNA), generates strong immunity with a single-dose, analogous to whole virus vaccines rather than a subunit vaccine, supporting a biological mechanism for single-dose efficacy rather than the prime-boost multi-dose schedule that optimizes sub-unit vaccine efficacy^14^. Single-dose HPV vaccination efficacy is equivalent to the licensed two- or three-dose regimen in randomized trials and observational studies^15–18^. Thus, in April 2022, the WHO recommended one or two doses of HPV vaccines for children, adolescents, and young adults aged 9–20 years. While more than 90 countries have adopted single-dose vaccination, some national guidelines continue to recommend multidose strategies^12^. Evidence for the durability of single-dose HPV vaccination and the impact of vaccinating persons older than 15 years potentially with prevalent HPV infection is needed, specifically as these infections acquired in early adulthood cause disease in midlife^19–22^.

This study evaluated zero versus single-dose HPV vaccination and used a blinded crossover design to evaluate (1) VE durability at 54 months, and (2) Vaccine effectiveness for a cohort 15–23 years, including those with prevalent infection at the time for vaccination. Thirty-six months after vaccination, bivalent and nonavalent vaccine efficacy was 98% for HPV 16/18, and nonavalent VE was 96% for HPV 16/18/31/33/45/52/58^23^. We hypothesized that single-dose HPV vaccine efficacy would be durable over 54 months, based on observed sustained antibody levels observed over 16 years in the observational Costa Rica Vaccine Trial, reflecting ongoing VE^24^. Further, we postulated that after infections prevalent at baseline clear, antibody protection against subsequent infection would be robust based on observational evidence for VE^25^. We evaluated VE by including persons with HPV infection at vaccination to provide insight into population level effectiveness of single-dose HPV vaccination.

## Results

### Study cohort

**Fig. 1:**
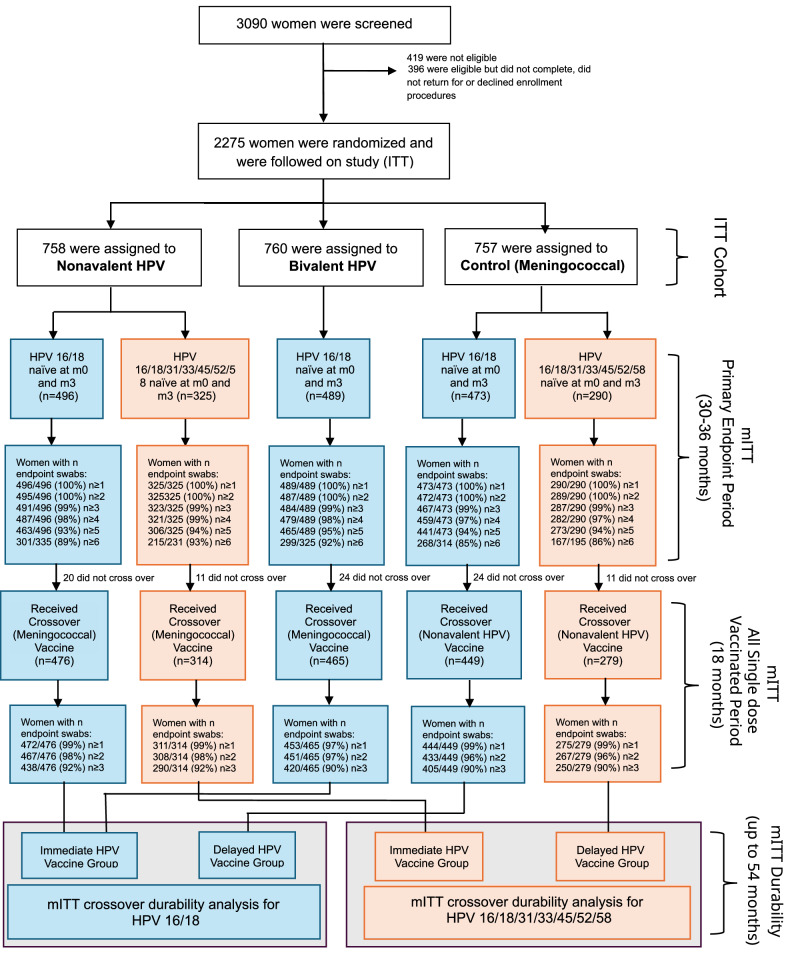
Randomized crossover trial profile. CONSORT diagram for the disposition of KEN SHE Study participants, including mITT cohort and vaccine group disposition for HPV 16/18 and HPV 16/18/31/33/45/52/58 durability analyses.

Between December 20th, 2018, and November 15th, 2019, 2,275 healthy participants were enrolled and randomly allocated: 758 to the nonavalent HPV vaccine group, 760 to the bivalent HPV vaccine group, and 757 to the control vaccine group (Fig. 1). Sixty-four percent (*N* = 1458) participants were HPV naïve and met criteria for the primary HPV 16/18 modified intention-to-treat (mITT) cohort; 496 were in the nonavalent, 489 in the bivalent, and 473 in the control group. Six hundred and fifteen participants were eligible for the HPV 16/18/31/33/45/52/58 mITT cohort, 325 were in the nonavalent, and 290 in the control vaccine group.

The participant characteristics between the three groups was comparable at each time point: enrollment and crossover vaccination (Table 1a, b). For the HPV 16/18 and 16/18/31/33/45/52/58 mITT cohorts, at crossover vaccination three years later, participants were older (median age = 20 years (interquartile range (IQR) 19–21 years)) compared to cohort at enrollment, where median age was 17 years. At crossover vaccination, 86% of participants reported one current main or steady partner compared with 69% at enrollment. Reflecting this change, non-vaccine HPV types and gonorrhea prevalence was higher at the cross-over vaccine visit (prevalence of non-vaccine HPV types was 52% and gonorrhea was 2.9% at crossover in the HPV 16/18 mITT cohort) compared to the enrollment vaccine visit (prevalence of non-vaccine HPV types was 37% and gonorrhea was 1.6% at enrollment). Chlamydia prevalence remained steady at both time points across the study groups at 11%.

**Table 1:** Characteristics of the mITT cohorts at enrollment and crossover vaccination visits

a. HPV 16/18 mITT Cohort									
		HPV 16/18 mITT							
		Enrollment vaccination visit				Crossover vaccination visit			
		Nonavalent HPV	Bivalent HPV	Control	All	Nonavalent HPV	Bivalent HPV	Control	All
**Characteristic**	**Category**								
Enrolled (*n*)	Total	496	489	473	1458	476	465	449	1390
Crossover visit	Month 30	–	–	–	–	141 (29.6%)	145 (31.2%)	138 (30.7%)	424 (30.5%)
	Month 36	–	–	–	–	335 (70.4%)	320 (68.8%)	311 (69.3%)	966 (69.5%)
Time to crossover (mo.)	Median	–	–	–	–	36	36	36	36
	IQR					(31, 36)	(31, 36)	(31, 36)	(31, 36)
Age at vaccination (y)	15–17	299 (60.3%)	278 (56.9%)	278 (58.8%)	855 (58.6%)	19 (4.0%)	16 (3.4%)	14 (3.1%)	49 (3.5%)
	18–20	197 (39.7%)	211 (43.1%)	195 (41.2%)	603 (41.4%)	278 (58.4%)	265 (57.0%)	259 (57.7%)	802 (57.7%)
	21–24	–	–	–	–	179 (37.6%)	184 (39.6%)	176 (39.2%)	539 (38.8%)
	Median	17	17	17	17	20	20	20	20
	IQR	(16, 18)	(16, 19)	(16, 19)	(16, 19)	(19, 21)	(19, 21)	(19, 21)	(19, 21)
Has a current main or steady sexual partner	No	144 (29.0%)	152 (31.1%)	145 (30.7%)	441 (30.2%)	60 (12.6%)	53 (11.4%)	66 (14.7%)	179 (12.9%)
	Yes	352 (71.0%)	337 (68.9%)	328 (69.3%)	1017 (69.8%)	416 (87.4%)	411 (88.4%)	381 (84.9%)	1208 (86.9%)
Condom use with last vaginal sex	No	153 (30.8%)	155 (31.7%)	140 (29.6%)	448 (30.7%)	265 (55.7%)	242 (52.0%)	232 (51.7%)	739 (53.2%)
	Yes	237 (47.8%)	235 (48.1%)	238 (50.3%)	710 (48.7%)	126 (26.5%)	138 (29.7%)	128 (28.5%)	392 (28.2%)
	No sex	106 (21.4%)	99 (20.2%)	95 (20.1%)	300 (20.6%)	85 (17.9%)	84 (18.1%)	87 (19.4%)	256 (18.4%)
*Chlamydia trachomatis*	Negative	438 (88.3%)	434 (88.8%)	413 (87.3%)	1285 (88.1%)	402 (84.5%)	399 (85.8%)	384 (85.5%)	1185 (85.3%)
	Positive	58 (11.7%)	55 (11.2%)	60 (12.7%)	173 (11.9%)	60 (12.6%)	48 (10.3%)	44 (9.8%)	152 (10.9%)
	Not done	0 (0.0%)	0 (0.0%)	0 (0.0%)	0 (0.0%)	14 (2.9%)	17 (3.7%)	20 (4.5%)	51 (3.7%)
*Neisseria gonorrhoeae*	Negative	488 (98.4%)	480 (98.2%)	466 (98.5%)	1434 (98.4%)	444 (93.3%)	435 (93.5%)	417 (92.9%)	1296 (93.2%)
	Positive	8 (1.6%)	9 (1.8%)	7 (1.5%)	24 (1.6%)	18 (3.8%)	12 (2.6%)	11 (2.4%)	41 (2.9%)
	Not done	0 (0.0%)	0 (0.0%)	0 (0.0%)	0 (0.0%)	14 (2.9%)	17 (3.7%)	20 (4.5%)	51 (3.7%)
Any non-vaccine HPV type DNA	Negative	317 (63.9%)	293 (59.9%)	307 (64.9%)	917 (62.9%)	218 (45.8%)	218 (46.9%)	229 (51.0%)	665 (47.8%)
	Positive	179 (36.1%)	196 (40.1%)	166 (35.1%)	541 (37.1%)	258 (54.2%)	247 (53.1%)	220 (49.0%)	725 (52.2%)
	Not done	0 (0.0%)	0 (0.0%)	0 (0.0%)	0 (0.0%)	0 (0.0%)	0 (0.0%)	0 (0.0%)	0 (0.0%)

b. HPV 16/18/31/33/45/52/58 mITT Cohort							
		HPV 16/18/31/33/45/52/58 mITT					
		Enrollment vaccination visit			Crossover vaccination visit		
		Nonavalent HPV	Control	All	Nonavalent HPV	Control	All
Characteristic	Category						
Enrolled (*n*)	Total	325	290	615	314	279	593
Crossover visit	Month 30	–	–	–	83 (26.4%)	86 (30.8%)	169 (28.5%)
	Month 36	–	–	–	231 (73.6%)	193 (69.2%)	424 (71.5%)
Time to crossover vaccination (mo.)	Median	–	–	–	36	36	36
	IQR	–	–	–	(32, 36)	(30, 36)	(31, 36)
Age at vaccination (y)	15–17	197 (60.6%)	168 (57.9%)	365 (59.3%)	11 (3.5%)	10 (3.6%)	21 (3.5%)
	18–20	128 (39.4%)	122 (42.1%)	250 (40.7%)	186 (59.2%)	159 (57.0%)	345 (58.2%)
	21–24	–	–	–	117 (37.3%)	110 (39.4%)	227 (38.3%)
	Median	17	17	17	20	20	20
	IQR	(16, 18)	(16, 19)	(16, 18)	(19, 21)	(19, 21)	(19, 21)
Has a current main or steady sexual partner	No	98 (30.2%)	95 (32.8%)	193 (31.4%)	35 (11.1%)	51 (18.3%)	86 (14.5%)
	Yes	227 (69.8%)	195 (67.2%)	422 (68.6%)	279 (88.9%)	228 (81.7%)	507 (85.5%)
Condom use with last vaginal sex	No	98 (30.2%)	78 (26.9%)	176 (28.6%)	167 (53.2%)	134 (48.0%)	301 (50.8%)
	Yes	156 (48.0%)	144 (49.7%)	300 (48.8%)	96 (30.6%)	83 (29.7%)	179 (30.2%)
	No sex	71 (21.8%)	68 (23.4%)	139 (22.6%)	51 (16.2%)	62 (22.2%)	113 (19.1%)
*Chlamydia trachomatis*	Negative	293 (90.2%)	252 (86.9%)	545 (88.6%)	273 (86.9%)	237 (84.9%)	510 (86.0%)
	Positive	32 (9.8%)	38 (13.1%)	70 (11.4%)	33 (10.5%)	30 (10.8%)	63 (10.6%)
	Not done	0 (0.0%)	0 (0.0%)	0 (0.0%)	8 (2.5%)	11 (3.9%)	19 (3.2%)
*Neisseria gonorrhea*	Negative	322 (99.1%)	285 (98.3%)	607 (98.7%)	299 (95.2%)	259 (92.8%)	558 (94.1%)
	Positive	3 (0.9%)	5 (1.7%)	8 (1.3%)	7 (2.2%)	8 (2.9%)	15 (2.5%)
	Not done	0 (0.0%)	0 (0.0%)	0 (0.0%)	8 (2.5%)	11 (3.9%)	19 (3.2%)
Any non-vaccine HPV type DNA	Negative	220 (67.7%)	208 (71.7%)	428 (69.6%)	148 (47.1%)	151 (54.1%)	299 (50.4%)
	Positive	105 (32.3%)	82 (28.3%)	187 (30.4%)	166 (52.9%)	128 (45.9%)	294 (49.6%)
	Not done	0 (0.0%)	0 (0.0%)	0 (0.0%)	0 (0.0%)	0 (0.0%)	0 (0.0%)

*IQR* interquartile range, *mo.* months, *y* years. For condom use with last vaginal sex, no sex is defined using the past year for enrollment visit data, and the past 6 months at the crossover visit. Any non-vaccine HPV type DNA is defined as HPV 26/35/39/40/42/43/44/51/53/54/56/59/61/66/68/69/70/73/82 type DNA positive in cervical swabs. Two participants are missing lab results for *Chlamydia trachomatis* and *Neisseria gonorrhea* at the crossover vaccination visit.

### Durability of VE

Ninety-five percent of participants in the HPV 16/18 mITT cohort (1390/1458) and 96% of participants in the HPV 16/18/31/33/45/52/58 mITT cohort (593/615) received crossover vaccination (Fig. 1). All mITT cohort participants received the assigned vaccination at crossover. In the HPV 16/18 mITT cohort, 21/1390 (1.5%) participants did not contribute follow-up time after crossover, and in the HPV 16/18/31/33/45/52/58 mITT cohort, 7/593 (1.2%) did not contribute post-crossover follow-up time. Overall, participants contributed a median of 53 months of follow-up time between December 2018 and the crossover month 18 data cut in June 2024. Retention for two or more swabs collected after crossover vaccination was 97% and 91% for three swabs (Tables S1–4). Of the endpoint swabs, 93% of swabs were cervical, and 7% of swabs were self-collected vaginal swabs.

**Fig. 2:**
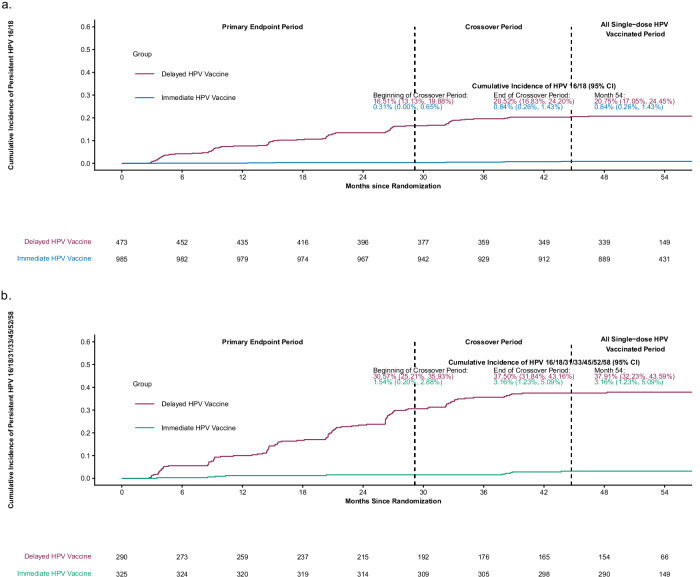
Cumulative incidence curves for the incidence of persistent HPV in the modified-intention-to-treat cohorts (durability analysis). Cumulative incidence curves were computed by vaccine group using Kaplan–Meier methods. The Crossover Period is defined at the study level, and spans from the first study day a participant received crossover vaccine to the last study day a participant received crossover vaccine. The Primary Endpoint Period includes follow-up from randomization until the date the first participant received crossover vaccine, and the All Single-dose HPV Vaccinated Period includes follow-up after the last day a participant received crossover vaccine. To assist with visualization, the *y*-axis (cumulative incidence) is shown 0 to 0.6. **a** Cumulative incidence of persistent HPV 16/18 in the HPV 16/18 mITT cohort (*n* = 1458 participants). The delayed HPV vaccine group includes participants randomized to the control (meningococcal) vaccine group who received nonavalent HPV vaccine at crossover, and the immediate vaccine group includes participants originally randomized to the nonavalent or bivalent vaccine groups and who received control vaccine at crossover. Sixty-four participants did not receive crossover vaccine and do not contribute follow-up time in the All Single-dose HPV Vaccinated Period. **b** Cumulative incidence of persistent HPV 16/18/31/33/45/52/58 in the HPV 16/18/31/33/45/52/58 mITT cohort (*n* = 615 participants). The delayed HPV vaccine group includes participants randomized to the control (meningococcal) vaccine group who received nonavalent HPV vaccine at crossover, and the immediate vaccine group includes participants originally randomized to the nonavalent vaccine group who received control vaccine at crossover. Twenty-two participants did not receive crossover vaccine and do not contribute follow-up time in the All Single-dose HPV Vaccinated Period.

For the primary outcome of incident persistent vaccine-type specific HPV, in the pre-crossover vaccination period, a total of 93 incident persistent infections were detected in the HPV 16/18 mITT cohort: one among the nonavalent vaccine group (incidence of persistent HPV 16/18 was 0.07/100 woman-years (95% CI: 0.002, 0.401)), three among participants assigned to the bivalent vaccine group (incidence of persistent HPV 16/18 was 0.22/100 woman-years (95% CI: 0.045, 0.641), and 89 among those assigned to the control vaccine group (incidence of persistent HPV 16/18 was 7.49/100 woman-years (95% CI: 6.02, 9.22)) (Table 2a and Fig. 2a). Post-crossover, 11 total incident persistent infections were detected in the HPV 16/18 mITT cohort: three among the nonavalent vaccine group, one among the bivalent vaccine group, and seven among participants assigned to the control vaccine group (Table 2a). Post-crossover, the incidence of persistent HPV 16/18 was 0.40/100 woman-years (95% CI: 0.083, 1.17) in the nonavalent vaccine, and 0.14/100 woman-years (95% CI: 0.004, 0.774) in the bivalent, compared to 1.26/100 woman-years (95% CI: 0.51, 2.59) in the control vaccine group. In the sensitivity analysis, when participants with evidence of HPV 16/18 infection detected at the time of crossover vaccination and 3 months post-crossover vaccination were excluded, no cases of persistent HPV 16/18 were observed in the control vaccine group post-crossover (Supplementary Table 8).

**Table 2:** Incidence of persistent HPV pre- and post- participant crossover vaccine receipt

a. HPV 16/18 mITT												
	Pre-participant crossover							Post-participant crossover				
					95% Confidence interval						95% Confidence interval	
Randomized group	HPV 16/18 naive at baseline (mITT) (*n*)	Incident persistent HPV 16/18 (*n*)	Woman-years of Follow-up	Incidence of persistent HPV 16/18 per 100 Woman-years	Lower bound	Upper bound	N at Risk	Incident persistent HPV 16/18 (*n*)	Woman-years of Follow-up	Incidence of persistent HPV 16/18 per 100 Woman-years	Lower bound	Upper bound
Nonavalent HPV	496	1	1390.96	0.072	0.002	0.401	471	3	749.65	0.400	0.083	1.17
Bivalent HPV	489	3	1368.59	0.219	0.045	0.641	450	1	719.94	0.139	0.004	0.774
Control	473	89	1187.76	7.49	6.02	9.22	360	7	557.43	1.26	0.505	2.59

b. HPV 16/18/31/33/45/52/58 mITT												
	Pre-participant Crossover							Post-participant crossover				
					95% Confidence interval						95% Confidence interval	
Randomized group	HPV 16/18/31/ 33/45/52/ 58 naive at baseline (mITT) (*n*)	Incident persistent HPV 16/18/31/33/45/52/58 (*n*)	Woman-years of Follow-up	Incidence of persistent HPV 16/18/31/33/45/52/58 per 100 Woman-years	Lower bound	Upper bound	*N* at risk	Incident persistent HPV 16/18/31/33/45/52/58 (*n*)	Woman-years of Follow-up	Incidence of persistent HPV 16/18/31/33/45/52/58 per 100 Woman-years	Lower bound	Upper bound
Nonavalent HPV	325	5	908.99	0.550	0.179	1.28	306	5	480.35	1.04	0.338	2.43
Control	290	98	672.53	14.57	11.83	17.76	177	9	262.16	3.43	1.57	6.52

Incidence of persistent HPV by randomized vaccine group in the mITT cohorts. Pre-participant crossover follow-up time is computed among participants HPV naïve at baseline, from enrollment to first positive result of consecutive positive HPV DNA results for participants with an endpoint, or from enrollment to last HPV DNA result not meeting endpoint criteria prior to crossover vaccine receipt for participants with no endpoint. For participants with an endpoint, result day is set to the mid-point between the first positive swab used to define the endpoint and the previous endpoint swab collection date. Post-participant crossover follow-up time is computed among participants HPV naïve at baseline who received crossover vaccine and who were still at risk for HPV (not previously censored and contributed at least one post-crossover swab), from date of crossover vaccine receipt to first positive result of consecutive positive HPV DNA results for participants with an endpoint, and from date of crossover vaccine receipt to last negative HPV DNA result for participants with no endpoint. For participants with an endpoint, result day is set to the mid-point between the first positive swab used to define the endpoint and the previous endpoint swab collection date. Exact 95% confidence interval for incidence rate computed using the Poisson distribution. **a** Pre- and post-participant crossover incident persistent HPV 16/18 in the HPV 16/18 mITT cohort (*n* = 1458). **b** Pre- and post-participant crossover incident persistent HPV 16/18/31/33/45/52/58 in the HPV 16/18/31/33/45/52/58 mITT cohort (*n* = 615).

Pre-crossover vaccination, a total of 103 incident persistent infections were detected in the HPV 16/18/31/33/45/52/58 mITT cohort: five among the nonavalent vaccine group, and 98 among participants in the control vaccine group (Table 2b). Pre-crossover, the incidence of persistent HPV 16/18/31/33/45/52/58 was 0.55/100 woman-years (95% CI: 0.179, 1.28) in the nonavalent group compared to 14.57/100 woman-years (95% CI: 11.8, 17.8) in the vaccine control group. Post-crossover, 14 total incident persistent infections were detected in the HPV 16/18/31/33/45/52/58 mITT cohort: five among participants assigned to the nonavalent vaccine group and nine in the control vaccine group (Table 2b). Post-crossover, the incidence of persistent HPV 16/18/31/33/45/52/58 was 1.04/100 woman-years (95% CI: 0.338, 2.43) in the nonavalent vaccine compared to 3.43/100 woman-years (95% CI: 1.57, 6.52) in the control vaccine control group. In the sensitivity analysis, when participants with evidence of HPV 16/18/31/33/45/52/58 infection detected at the time of crossover vaccination and 3 months post vaccination were excluded, the post-crossover incidence of persistent HPV 16/18/31/33/45/52/58 was 0.54/100 woman-years (95% CI: 0.01, 3.03) in the control vaccine group (Supplementary Table 8).

**Fig. 3:**
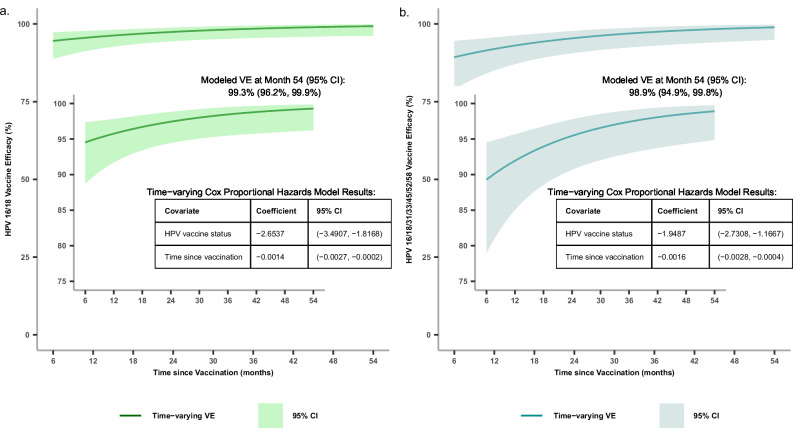
Vaccine efficacy to prevent incident persistent HPV as a function of time in the modified-intention-to-treat cohorts (durability analysis). HPV vaccine efficacy (VE) as a function of time computed using a Cox proportional hazards regression model with time-varying covariates for HPV vaccine status and time since HPV vaccination. VE is plotted over time since vaccination (solid line) with 95% confidence intervals (shaded bands) around VE at each timepoint. Data are shown at full (100%) scale and as an inset with the *y*-axis (VE) zoomed in to 75–100%. **a** HPV VE as a function of time for incident persistent HPV 16/18 in the HPV 16/18 mITT cohort (*n* = 1458 participants). **b** HPV VE as a function of time for incident persistent HPV 16/18/31/33/45/52/58 in the HPV 16/18/31/33/45/52/58 mITT cohort (*n* = 615 participants).

In the HPV 16/18 mITT cohort, the cumulative incidence of HPV 16/18 in the immediate HPV 16/18 vaccine group was 0.31% (95% CI: 0.00, 0.65%) pre-crossover, 0.84% (95% CI: 0.26, 1.43%) at the end of the crossover period, and 0.84% (95% CI: 0.26, 1.43%) at month 54 (Fig. 2a). The HPV 16/18 cumulative incidence in the delayed vaccine group was 16.51% (95% CI: 13.13, 19.88%) pre-crossover, 20.52% (95% CI: 16.83, 24.20%) at the end of the crossover period, and 20.75% (95% CI: 17.05, 24.45%) at month 54. The incidence of HPV 16/18 post-crossover in the delayed vaccine group was 1.26/100 woman-years (95% CI: 0.51, 2.59). The incidence in the immediate vaccine group was 0.27/100 woman-years (95% CI: 0.07, 0.70) (Supplementary Fig. 1). For HPV 16/18, VE as a function of time since vaccination was estimated as 99.3% (95% CI: 96.2, 99.9%) at 54 months with a coefficient for time since vaccination of −0.0014 (95% CI: −0.0027, −0.0002) (Fig. 3a).

In the HPV 16/18/31/33/45/52/58 mITT cohort, the cumulative incidence of HPV 16/18/31/33/45/52/58 in the immediate HPV vaccine group was 1.54% (95% CI: 0.20, 2.88%) pre-crossover, 3.16% (95% CI: 1.23, 5.09%) at the end of the crossover period, and 3.16% (95% CI: 1.23, 5.09%) at month 54 (Fig. 2b). The HPV 16/18/31/33/45/52/58 cumulative incidence in the delayed vaccine group was 30.57% (95% CI: 25.21, 35.93%) pre-crossover, 37.50% (95% CI: 31.84, 43.16%) at the end of the crossover period, and 37.91% (95% CI: 32.23, 43.59%) at month 54. The incidence of HPV 16/18/31/33/45/52/58 post-crossover in the delayed vaccine group was 3.43/100 woman-years (95% CI: 1.57, 6.52). The post-crossover incidence in the immediate vaccine group was 1.04/100 woman-years (95% CI: 0.34, 2.43) (Supplementary Fig. S2). Nonavalent VE as a function of time since vaccination was estimated as 98.9% (95% CI: 94.9, 99.8%) at month 54 with a coefficient for time since vaccination of −0.0016 (95% CI: −0.0028, −0.0004) (Fig. 3b). We conducted a sensitivity analysis excluding the baseline serology information for the mITT HPV 16/18 and HPV 16/18/31/33/45/52/58 cohorts and found no meaningful difference in VE (Supplementary Table 11).

### Background HPV exposure

Incident persistent non-vaccine HPV types, an unplanned exploratory outcome, was comparable between the study groups within and between the pre-crossover and post-crossover time periods. In the pre-crossover vaccination period, the incidence of persistent non-vaccine HPV types was 25.8/100 woman-years (95% CI: 22.4, 29.6) in the immediate HPV vaccination group and 23.2/100 woman-years (95% CI: 18.8, 28.3) in the delayed vaccination group; and in the post-crossover vaccination period incidence was 22.7/100 woman-years (95% CI: 16.7, 30.0) in the immediate HPV vaccine and 20.2/100 woman-years (95% CI: 12.8, 30.3) in the delayed group (Supplementary Table 5).

### Vaccine effectiveness

**Fig. 4:**
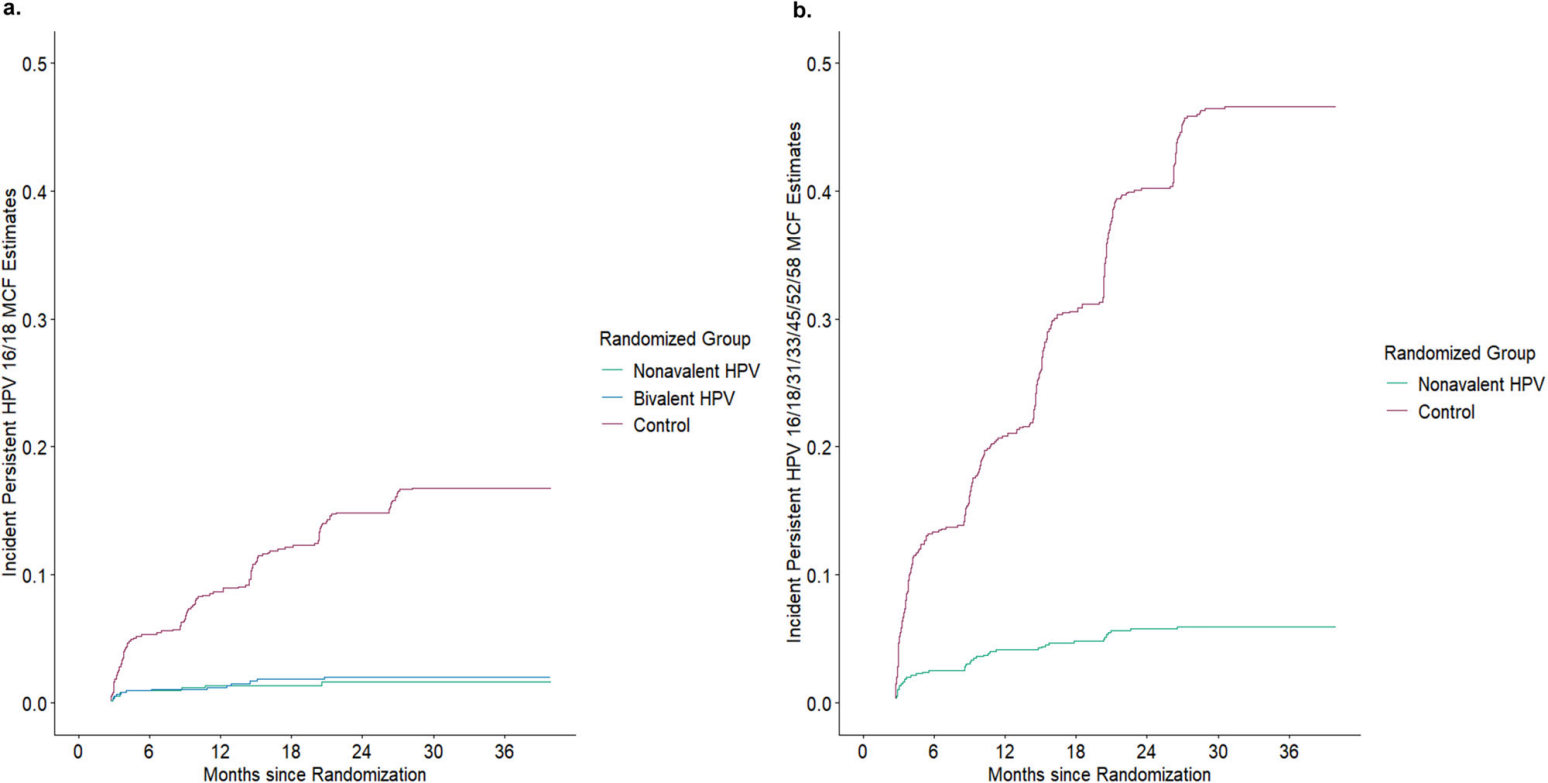
Nelson–Aalen cumulative hazard curves for incident persistent HPV events in the intention-to-treat cohort (vaccine effectiveness analysis). Mean cumulative function (MCF) estimates for incident persistent HPV events by time since randomization in the ITT cohort, using the Nelson–Aalen estimator of the cumulative hazard rate function. Each participant may contribute more than one incident persistent HPV event of different HPV types. Time at risk computed using endpoint swabs collected up to an including the crossover visit. **a** Cumulative hazard curves for incident persistent HPV 16/18 events (*n* = 2275 participants). **b** Cumulative hazard curves for incident persistent HPV 16/18/31/33/45/52/58 events (*n* = 1515 participants).

In the pre-planned exploratory analysis, the intention-to-treat (ITT) vaccine effectiveness was assessed between the randomly allocated groups at month 36, the period during which a placebo-equivalent control group was assessable. In the HPV 16/18 ITT cohort, there were a total of 173 prevalent persistent HPV 16/18 infections at enrollment: 49 among participants assigned to the nonavalent group, 56 in bivalent group, and 68 among those assigned to the control vaccine group (Table 3a). Of those prevalent infections, 14, 19, and 13, in the nonavalent, bivalent, and control groups, respectively, did not meet the criteria for clearance—two consecutive negative type-specific HPV DNA tests at least four months apart. Over 36 months, there were 12 incident persistent HPV 16/18 infections in the nonavalent group, 15 in the bivalent group, and 125 in the control group. HPV 16/18 incidence was 0.58 per 100 woman-years in the nonavalent, 0.72 per 100 woman-years in the bivalent vaccine group, and 6.00 per 100 woman-years in the control group; nonavalent vaccine effectiveness was 90.37% (*p* < 0.0001) and bivalent vaccine effectiveness was 88.08% (*p* < 0.0001). The Nelson–Aalen cumulative hazard curves for incident persistent HPV 16/18 events in the ITT cohort (Fig. 4a) shows clear separation of the intervention and control groups over 36 months.

**Table 3:** Incidence of total persistent HPV infections and vaccine effectiveness, by randomized group (ITT cohort, primary endpoint period)

a. HPV 16/18												
							95% Confidence interval		Statistical comparisons			
Randomized group	Enrolled (*n*)	Total persistent HPV 16/18 infections at enrollment (*n*)	Did not have 2 consecutive negative results during follow-up (*n*)	Total Incident Persistent HPV 16/18 Infections (*n*)	Woman-years of Follow-up	Incidence of persistent 16/18 Infections per 100 Woman-years	Lower bound	Upper bound	Comparison	Vaccine effectiveness	95% CI	*P*-value
Nonavalent HPV	758	49	14	12	2077.60	0.58	0.30	1.01	Nonavalent HPV v. Control	90.37%	(82.6%, 94.7%)	<0.0001
Bivalent HPV	760	56	19	15	2097.12	0.72	0.40	1.18	Bivalent HPV v. Control	88.08%	(79.6%, 93.0%)	<0.0001
Control	757	68	13	125	2083.53	6.00	4.99	7.15				

b. HPV 16/18/31/33/45/52/58												
							95% Confidence interval		Statistical comparisons			
Randomized group	Enrolled (*n*)	Total persistent HPV 16/18/31/33/45/52/ 58 infections at enrollment (*n*)	Did not have 2 consecutive negative results during follow-up (*n*)	Total incident persistent HPV 16/18/31/33/45/52/58 infections (*n*)	Woman-years of follow-up	Incidence of persistent 16/18/31/33/45/52/ 58 infections per 100 woman-years	Lower bound	Upper bound	Comparison	Vaccine effectiveness	95% CI	*P*-value
Nonavalent HPV	758	135	48	44	2077.60	2.12	1.54	2.84	Nonavalent HPV v. Control	87.25%	(82.5%, 90.7%)	<0.0001
Control	757	164	35	346	2083.53	16.61	14.90	18.45				

Incidence of persistent HPV infections by randomized vaccine group in the intention-to-treat cohort. Woman-years of follow-up time is computed from randomization to last pre-crossover endpoint swab. Nineteen participants did not contribute a post-randomization endpoint swab. Each participant may contribute more than one incident persistent HPV infection of different HPV types. Incidence rate ratio (IRR) and corresponding 95% confidence intervals are estimated using a single Poisson regression model with a class variable for vaccine arm as the only covariate and an offset of log(woman-years). Vaccine Effectiveness is computed as 100*(1–IRR). Standard errors from the Poisson models were used to generate Z-scores against the null hypothesis of IRR = 1.0 (2253 degrees of freedom) and compute corresponding two-sided *p*-values. **a** Incidence of persistent total HPV 16/18 infections and vaccine effectiveness in the ITT cohort (*n* = 2275 participants). Time a participant is persistent positive for both HPV 16 and HPV 18 is included as woman-years of follow-up. **b** Incidence of total persistent HPV 16/18/31/33/45/52/58 infections and vaccine effectiveness in the ITT cohort nonavalent HPV and control vaccine groups (*n* = 1515 participants).

In the HPV 16/18/31/33/45/52/58 ITT cohort, there were a total of 299 HPV 16/18/31/33/45/52/58 prevalent persistent infections at enrollment: 135 among participants assigned to the nonavalent group and 164 among those assigned to the control vaccine group (Table 3b). Of those prevalent infections, 48 and 35, in the nonavalent and control groups, respectively, did not meet the criteria for clearance. Over 36 months, there were 44 incident persistent HPV 16/18/31/33/45/52/58 infections in the nonavalent group and 346 in the control group. HPV 16/18/31/33/45/52/58 incidence was 2.12 per 100 woman-years in the nonavalent vaccine group and 16.61 per 100 woman-years in the control group; nonavalent vaccine effectiveness was 87.25% (*p* < 0.0001). In a subgroup analysis, with subgroups defined by a positive DNA result in the enrollment cervical swab only (vaginal swab DNA and antibody results not used), nonavalent vaccine effectiveness among participants positive for HPV 16/18/31/33/45/52/58 was 80.61% (Supplementary Table 10). The Nelson–Aalen cumulative hazard curves for incident persistent HPV 16/18/31/33/45/52/58 events in the ITT cohort (Fig. 4b) shows graphically the difference in incident cases in the study groups.

Serious adverse events (SAEs) were experienced by 330 participants over 54 months, which included 227 participants with pregnancy-related SAEs, 94 with infections or inflammatory conditions, eight injuries, and 20 mental health illnesses. There were zero vaccine-associated SAEs. Overall, the SAE frequency was similar between groups (Table 4). There were five deaths in the study due to unsafe abortion and sepsis, suicide, hepatocellular carcinoma, complications following an emergency cesarean section for fetal distress, and one unknown cause with brief symptoms of cough productive of bloody sputum. From the cytological evaluation conducted at the vaccination visit, eight participants had high-grade cervical cytology findings and were followed until the lesions resolved or the participant received treatment. Social harms were reported by 0.44% of participants (*n* = 10), including partner physical and verbal abuse and lack of social support from friends and family for trial participation.

**Table 4:** Participants experiencing adverse events (ITT)

	Randomized group			
	Nonavalent HPV	Bivalent HPV	Control	All
Enrolled, *n*	758	760	757	2275
Any SAE, *n*(%)	104 (13.7%)	109 (14.3%)	117 (15.5%)	330 (14.5%)
Any pregnancy-related, *n*(%)	81 (10.7%)	71 (9.3%)	75 (9.9%)	227 (10.0%)
Any infection/inflammation, *n*(%)	21 (2.8%)	33 (4.3%)	40 (5.3%)	94 (4.1%)
Any injury, *n*(%)	0 (0.0%)	4 (0.5%)	4 (0.5%)	8 (0.4%)
Any mental health, *n*(%)	4 (0.5%)	9 (1.2%)	7 (0.9%)	20 (0.9%)

Participants may have more than one event across categories. No vaccine-related SAEs were reported.

## Discussion

Four and a half years following vaccine administration, both single-dose bivalent and nonavalent HPV vaccines continue to demonstrate sustained and durable high efficacy against vaccine-specific oncogenic HPV infections without evidence of waning. Estimated VE remained ≥99% for the bivalent and nonavalent vaccines against HPV types 16 and 18; and exceeded 98% for the nonavalent vaccine against HPV types 16, 18, 31, 33, 45, 52, and 58—types responsible for approximately 75% and 95% globally and 72% and 92% of cervical cancers in Africa, respectively^26^. Importantly, this high efficacy persisted as the median age at vaccination increased from 17 to 20 years, remaining robust from ages 18 through 23 years. The high vaccine effectiveness observed in the ITT cohort (90%), which included individuals already infected with HPV at vaccination, highlights the substantial potential public health impact, particularly in regions experiencing pronounced disparities in cervical cancer incidence and mortality. In the ITT cohort, we observed that once infections present at the time of vaccination cleared, which occurred in 73% (127/173) of infections at enrollment, effectiveness was high. Among persons with prevalent cervical infection at the time of vaccination, vaccine effectiveness remained high (80%).

Critically, vaccine efficacy remained highly certain at levels of at least 95% against HPV types 16, 18, 31, 33, 45, 52, and 58, the lower bounds of confidence intervals, supporting this sustained protection over 54 months. Notably, the incidence rate of persistent HPV infection continued to increase steadily in the control group until crossover vaccination, at which point incidence rate declined to match that of the immediate vaccination group. Implementation of catch-up vaccination initiatives targeting adolescents and young adults aged 15–23, who fall outside the current vaccination guidelines, offers significant potential to reduce persistent oncogenic HPV infections. Importantly, empiric data are needed on VE of one-dose vaccination among HPV-negative adolescents and young women. The increase in non-vaccine HPV types and gonorrhea at the cross over vaccine visit compared to the enrollment visit likely reflects aging of the cohort into early adulthood and corresponding increases in exposure risk. Despite the increase in exposure, no difference was seen in VE for vaccine type-specific HPV. While the three-dose schedule was based on the hepatitis B subunit vaccines, a single-dose of the HPV vaccine has high and durable efficacy. Furthermore, a single-dose vaccination strategy could substantially enhance vaccine accessibility, increase overall coverage, and present a cost-effective approach to global cervical cancer prevention.

The study has several notable strengths, including its randomized, double-blind, controlled design; high participant retention; and the use of cervical HPV DNA to assess outcomes. The determination of persistent HPV infection and the head-to-head comparison of licensed bivalent and nonavalent HPV vaccines for protection against oncogenic HPV types further enhance the rigor of the findings. The extended duration of follow-up adds to the robustness of the efficacy estimates. Importantly, the trial successfully enrolled and retained individuals already exposed to HPV infection across all randomized groups, enabling a timely and meaningful assessment of single-dose vaccine efficacy.

Compared to the analysis at 36 months, vaccine efficacy estimates remained consistent through 54 months, with slightly higher point estimates and narrower confidence intervals. These improvements are likely due to the accrual of additional follow-up time beyond the six- to twelve-month window, which may have previously included baseline infections that were not detected at early follow-up visits. This additional follow-up period reduced the influence of such transient infections on efficacy estimates, leading to greater precision and stability in the results^20,21^.

The study has limitations, of which the absence of a control group post-crossover vaccination is notable. A crossover design inherently limits long-term comparisons because a contemporaneous unvaccinated control group is no longer available after crossover, requiring durability to be assessed using modeled estimates rather than direct placebo comparisons. Nonetheless, this design was ethically appropriate once efficacy was demonstrated and minimized attrition while still enabling robust evaluation of long-term vaccine performance. However, through both the observed incidence of persistent HPV infection and Cox regression analysis accounting for time-varying covariates, VE was estimated as a function of time. It is possible that the observed incidence in a control group would be different from the modeled incidence, but there was no change in HPV incidence of non-vaccine types, supporting this modeled approach. The mITT was not redefined at the cross-over vaccination; however, this was considered in a sensitivity analysis, which demonstrate high protection against persistent infection when prevalent infections at the time of vaccination were excluded. The median duration of follow-up of 53 months; however, observational data for single-dose HPV vaccination support efficacy over a decade^17^. We are collecting additional data in this cohort to protocol-specified, funded up to 90 months follow-up post-vaccination^27^. Further, the antibody plateau level for single-dose HPV vaccination is reached by month 12^15^, suggesting that we have observed steady state efficacy. While a small proportion of swabs were self-collected versus clinician-collected, the correlation between self-collected vaginal and provider-collected cervical swabs is high^28^ and there was no difference in the results when self-collected swabs were excluded.

Effective tools are needed to close gaps in disparities of cervical cancer incidence worldwide^1^. Cervical cancer is the largest contributor to cancer-related mortality of mid-adult women in southern and East Africa and is largely preventable with HPV vaccination prior to acquisition of the infection that in a small number of cases is oncogenic. Since most infections among young women clear, single-dose catch-up vaccination to the mid-20s is a feasible strategy to prevent cervical cancer in mid-life. In addition, 5% of all cancers globally are HPV-associated: HPV-associated head and neck cancer is the fastest-growing HPV-associated cancer in the United States. Scaling up single-dose HPV vaccination has the potential to avert not only cervical cancer morbidity and mortality globally but also could impact HPV-associated cancers more broadly. Here, we present robust evidence on the durability of single-dose HPV vaccine efficacy and effectiveness when persons with HPV infection at the time of vaccination are included in the analysis. Single-dose HPV vaccination could facilitate rapid scale-up of vaccination worldwide with the potential to avert HPV-associated disease.

## Methods

### Ethics statement

We conducted a clinical trial to test the efficacy of single-dose HPV vaccination among young women aged 15 and older within the context of cytological screening for dysplastic lesions. This was determined to be ethical, as vaccination for this age group in Kenya and many low human development index (HDI) countries is not currently supported through national programs or global immunization bodies^29^. Written informed consent was obtained from all participants.

The KEMRI Scientific and Ethics Review Unit (SERU) and the Massachusetts General Hospital (MGH) Institutional Review Board (IRB) approved the study. The study was registered with ClinicalTrials.gov (NCT03675256).

### Study design

This randomized, multicenter, double-blind, parallel, three-group controlled, crossover superiority trial tested the efficacy of single-dose bivalent (HPV 16/18) and single-dose nonavalent (HPV 16/18/31/33/45/52/58/6/11) HPV vaccination^30^. The study was conducted at three Kenya Medical Research Institute (KEMRI) clinical sites in Kisumu, Thika, and Nairobi. Recruitment was conducted through community outreach activities in surrounding communities, with interested individuals screened for eligibility at study clinics.

To expedite crossover vaccine receipt following the release of the primary results, participants were offered vaccination at their next study visit, which was at either month 30 or 36. Participants in the meningococcal vaccine group received the nonavalent HPV vaccine, and those in the nonavalent HPV and bivalent HPV vaccine groups received the meningococcal vaccine, maintaining the study blind through crossover vaccination (Fig. 1).

### Participants

At the time of enrollment, individuals were eligible if they self-reported being assigned female at birth, were between 15 and 20 years old (inclusive), had ever been sexually active with one to five lifetime sexual partners, and planned to remain in the study area for the duration of the trial. Exclusion criteria included living with human immunodeficiency virus (HIV) (due to limited data on the efficacy of single-dose HPV vaccination in this population), prior HPV vaccination, known allergy to vaccine components or latex, current pregnancy, history of hysterectomy, or a personal history of autoimmune, degenerative, or genetic disorders. Recruitment was conducted through community outreach. To be eligible for continued follow-up in the KEN SHE Study, participants were required to be currently enrolled and provide written informed consent to blinded crossover vaccination and follow-up. Participants could elect to receive crossover vaccination without follow-up.

### Randomization and masking

Meningococcal vaccine was selected as the control because it may offer clinical benefits while having no effect on HPV-related outcomes. Participants were randomly assigned to one of three groups: (1) immediate nonavalent HPV vaccination (Gardasil-9®) with delayed meningococcal vaccination; (2) immediate bivalent HPV vaccination (Cervarix®) with delayed meningococcal vaccination; or (3) immediate meningococcal vaccination with delayed nonavalent HPV vaccination. After the primary study results became available, participants received blinded crossover vaccination at their next scheduled visit (month 30 or 36).

An unblinded statistical analyst generated the original randomization sequence using SAS v9.4. Randomization was stratified by site, using a fixed block size of 15 and a 1:1:1 allocation. Blinded study assignment was implemented via http://www.randomize.net (Ottawa, ON, Canada). Study staff, participants, investigators, clinic staff, lab technicians, the endpoints adjudication committee members, and other study team members did not have access to the randomization codes, except for the unblinded statistical analysts and unblinded pharmacists at each site. The study blind was maintained through crossover vaccination. The unblinded pharmacists at each site received the information for the crossover vaccine for each participant and the corresponding blinded crossover vaccine ID. At the blinded crossover vaccination visit, an unblinded pharmacist recorded the participant identifier and crossover vaccine ID on an electronic case report form (eCRF), drew up the vaccine in a masked syringe, and administered the vaccination via the intramuscular route. An independent observer, not on the study team, observed the masked vaccination to assess the success of masking.

### Procedures

At the crossover vaccination visit, cervical swabs for HPV DNA testing were collected prior to crossover vaccination. Participants had study visits three months after crossover vaccination, six months after crossover, and then every six months for 18 months after crossover, equivalent to 48–54 months total study time. Providers administered clinical questionnaires and collected a cervical swab at each six-month visit. Participants self-collected vaginal swabs using validated instructions at month three; self-collected swabs, which have similar accuracy compared to provider-collected cervical swabs^28^, were available at subsequent follow-up visits by participant choice.

All participants received cytological screening for cervical cancer at enrollment, with repeat screening as indicated clinically and planned at 5 years. Sexual and reproductive health services (contraception, sexually transmitted infection diagnosis and treatment, and HIV pre-exposure prophylaxis) and mental health counseling were available at every visit. All questionnaires used eCRFs (DF/Net Research, Inc. ©, Seattle, WA, US).

### Laboratory methods

HPV DNA genotyping was conducted using the Anyplex II HPV28 or Allplex HPV28 assay (Seegene, Seoul, South Korea), multiplexed type-specific real-time polymerase chain reaction (PCR) based assays that detect 28 HPV types with high agreement^31,32^ at the University of Washington (UW) East Africa Sexually Transmitted Infections (STI) Laboratory, Mombasa, Kenya with standard proficiency testing^33^. For Anyplex II HPV28, HPV-positive samples, a low (+), intermediate (++), or high (+++) positivity was indicated; + or greater were considered positive. Allplex HPV28 provides Ct values which correlate well with Anyplex II signal intensity scores^34^. The assay runs included negative and positive controls, and the housekeeping human gene, β-globin, as an internal control. Runs were performed with CFX96 Real-time PCR System (BioRad, Hercules, California).

*Neisseria gonorrhoeae* and *Chlamydia trachomatis* were assessed by nucleic acid amplification testing (APTIMA; Hologic/GenProbe, San Diego, CA) at the UW East Africa STI Laboratory.

### Outcomes

The primary trial endpoint was incident persistent cervical vaccine type-specific HPV infection. Persistent HPV, a surrogate marker for cervical dysplasia/precancer used in HPV vaccine efficacy clinical trials, was defined as high-risk vaccine type-specific HPV (i.e., HPV 16/18 for the bivalent vaccine and HPV 16/18/31/33/45/52/58 for the nonavalent vaccine) detected at two consecutive time points after month three up to and including month 54, which were obtained no less than four months apart (with the same HPV type at both time points). Cervical swabs were tested for the primary endpoint; vaginal swabs were substituted if necessary. The durability analysis was planned for 18 months post-crossover vaccination, approximately study month 54.

Safety is reported according to local guidelines and was assessed through adverse event reporting following Division of Allergy and Infectious Disease (DAIDS) guidelines^35^. Participants were monitored for adverse events 30 min after crossover vaccination, asked about adverse events at each study visit, and reported adverse events outside of study visits. The study clinical monitor followed SAEs, including access to medical records. The study PI and clinical monitor reviewed investigator determined relationship of SAEs to vaccination. We followed SERU’s guidance for reporting SAEs. An independent Data Safety and Monitoring Board (DSMB) was constituted to review study progress, participant safety, and the primary outcome; the DSMB met annually.

### Statistical analysis

The mITT cohorts excluded participants with evidence of baseline HPV infection with the outcome HPV types and therefore estimates vaccine efficacy among those HPV-naïve at enrollment vaccination. The ITT cohort included all randomized participants regardless of baseline infection status and reflects vaccine effectiveness under real-world conditions where pre-vaccination HPV testing is not performed. Safety was assessed among all participants. We performed all analyses using SAS software, version 9.4 (SAS Institute, North Carolina, US) and R (version 4.2.2).

#### VE durability analysis

The pre-planned primary outcome of the study was incident, persistent vaccine-type specific HPV infection. Participants included in the month 54 durability analysis tested HPV DNA negative at enrollment and at month three, and HPV antibody negative at enrollment, comprising the mITT cohorts. For inclusion in the HPV 16/18 mITT cohort, participants were HPV 16/18 naïve at enrollment. Similarly, for the HPV 16/18/31/33/45/52/58 mITT cohort, participants were HPV 16/18/31/33/45/52/58 naïve at enrollment. Because persistent infections were captured through study monitoring, no further exclusion criteria were applied post-crossover. To determine HPV exposure over the duration of the study, we evaluated the incidence of persistent non-vaccine HPV types by study group across the pre- and post-crossover periods, an unplanned exploratory outcome of the study.

Month 54 HPV incidence and VE durability analyses were performed on the mITT cohorts. Incidence of persistent HPV per 100 woman-years is reported pre- and post-crossover vaccine receipt and by original randomized group in the HPV 16/18 mITT and the HPV 16/18/31/33/45/52/58 mITT cohorts. For the pre-crossover period, follow-up time was computed from the date of randomization to the midpoint between the date of the first positive result of an endpoint and the previous negative result for participants with an endpoint, or to the last timepoint not meeting endpoint criteria prior to crossover vaccine receipt for participants without an endpoint. Participants who had the first of two consecutive positive results at the crossover visit were considered to have an event in the pre-crossover period. Post-crossover period follow-up time was computed as the date of crossover vaccine receipt to the midpoint between the date of the first positive result of a post-crossover endpoint and the previous negative result, or to the last timepoint not meeting endpoint criteria for participants without an endpoint. A sensitivity analysis was designed to estimate post-crossover incidence to complement the primary VE analysis^16^ by subsetting to those participants with no evidence of HPV infection at or before three months post-crossover.

Cumulative incidence Kaplan–Meier curves of time to incident persistent infection were computed through month 54 by group. For the cumulative incidence of persistent HPV 16/18, since VE for HPV 16/18 was comparable between the original randomized bivalent and nonavalent vaccine groups, the two groups were combined into the immediate HPV 16/18 vaccine group and compared to the delayed HPV vaccine group. For the cumulative incidence of persistent HPV 16/18/31/33/45/52/58, the original randomized nonavalent vaccine group is the immediate vaccine group, and the randomized control group is the delayed HPV vaccine group. The crossover study period was defined as the period between the earliest and latest study timepoint at which a participant received crossover vaccination.

To estimate the durability of VE of the interventions versus control over the 54-month follow-up period, we used a Cox regression model with time-varying covariates for HPV vaccine status and time since HPV vaccination (both set to 0 pre-vaccination). Follow-up time for participants who did not have a persistent infection endpoint was calculated at the last study visit where they did not meet the criteria for persistent infection. If the vaccine is not durable (i.e., the vaccine effect wanes), we expect the coefficient for time since vaccination to be positive and statistically significantly different from zero, which would result in an estimate of VE that decreases over time. To help with interpretation, estimated vaccine efficacy was plotted over time since vaccination with 95% confidence intervals.

#### Vaccine effectiveness

Pre-planned exploratory vaccine effectiveness analyses were conducted among all randomized participants prior to crossover, extending the analysis cohort to include those with evidence of current or past HPV infection (HPV DNA positive at enrollment and/or month three and/or tested HPV antibody positive at enrollment); i.e., ITT cohort. To assess vaccine effectiveness in the ITT cohort, we used a Poisson regression model with an outcome of number of persistent infections and an offset of follow-up time to compare the incidence of total persistent HPV infections in the randomized HPV vaccine versus control groups over the 30–36-month follow-up period. All randomized participants were considered at risk for at least one persistent HPV vaccine type infection throughout follow-up, and a participant could contribute multiple incident persistent HPV infections of the same or different HPV type. Follow-up time is computed from randomization to the last endpoint swab at or prior to the crossover visit. Two consecutive HPV negative results of the previously persistent positive HPV type were required to meet the definition of HPV clearance, at which time participants were eligible to contribute new incident persistent infections of that HPV type. Vaccine effectiveness and 95% confidence intervals were computed from the incidence rate ratio (IRR) as [100*(1–IRR)]. Two-sided *p*-values were calculated using the standard errors against the null hypothesis for IRR = 1.0. Nelson–Aalen cumulative hazard curves of time to infection were calculated by intervention group. A sensitivity analysis was conducted among participants who were HPV positive at enrollment to estimate vaccine effectiveness among persons with cervical infection at the time of vaccination.

### Ethics and inclusion statement

Data for this study, including from Kenya, were collected via eCRFs in Kenya. Seven colleagues (M.A.O., E.A.B., B.N., I.W., C.B., S.K., and N.R.M.), including the senior author (M.A.O.), are from Kenya, a low-and-middle-income country, and one other (R.V.B.) is South African and is now based in a high-income country. Local authors were included in the study design, study implementation, data ownership, intellectual property, and authorship of publications throughout the study. The roles and responsibilities for study design, implementation, analysis, interpretation, and dissemination of results was agreed on ahead of the study. The Study Team conducted study training, writing workshops, and investigator meetings. At the International Papillomavirus Conference in 2024, seventeen investigators were first authors on abstracts presented from this body of work, including ten new investigators. We fully endorse and are committed to the Nature Portfolio journals’ guidance on low-and-middle-income country authorship and inclusion. This research is locally relevant to Kenya and other countries that have not achieved the 90% HPV vaccine coverage goal. In addition to the results presented in this paper, together with the Kenyan Ministry of Health, we conducted health economic analyses for the implementation of single-dose HPV vaccination, which has now been adopted as the primary strategy for HPV immunization in Kenya. The KEMRI SERU (nos. 3745 and 3741) and the Massachusetts General Hospital Institutional Review Board (no. 2022P001178) approved the study. Study participation may have carried stigmatization associated with vaccination. The data collection and analysis techniques employed raised no risks pertaining to incrimination, discrimination, animal welfare, the environment, health, safety, security, or other personal risks. All HPV and STI testing was conducted at local laboratories. Serum for specialized HPV antibody testing was shipped to Seattle for testing. No cultural artifacts or associated traditional knowledge has been transferred out of any country. In preparing the manuscript, the authors reviewed relevant studies from Kenya.

### Reporting summary

Further information on research design is available in the Nature Portfolio Reporting Summary linked to this article.

## Supplementary information

Supplementary Information

Reporting Summary

Transparent Peer Review file

## Data Availability

Data cannot be shared publicly because this study was conducted with approval from the Kenya Medical Research Institute (KEMRI) Scientific and Ethics Review Unit (SERU), which requires that data from studies (including de-identified data) are released only after SERU has provided written approval for additional analyses. A complete de-identified dataset sufficient to reproduce the study findings will be made available upon written request after approval from SERU. To request these data, please contact the KEN SHE Scientific Committee at lnakatsuka@partners.org.
