# Supplementary material for: Efficacy and durability of immediate versus delayed single-dose HPV vaccination for persistent infection among young women in Kenya: a randomized, blinded, cross-over clinical trial
Source: Nat Commun. 2026 May 11;17:6289. doi: 10.1038/s41467-026-72654-8 (PMC13377066; doi:10.1038/s41467-026-72654-8)
Supplement: Supplementary file 1 — Supplementary Information [file 41467_2026_72654_MOESM1_ESM.pdf]

**Efficacy and durability of immediate single-dose human papillomavirus vaccination versus delayed vaccination in persistent HPV infection among young women: a randomized, blinded, cross-over clinical trial**

# Table of Contents


Table of Contents ........ 1

Supplementary Information ........ 2

KEN SHE Study Team........ 2

Supplementary Tables ........ 3

Supplementary Table 1 | Completeness of Endpoint Swab* Collection, by Randomized Group (HPV 16/18 mITT Cohort) ........ 3

Supplementary Table 2 | Number of Endpoint Swabs per Participant, by Randomized Group (HPV 16/18 mITT Cohort) ........ 4

Supplementary Table 3 | Completeness of Endpoint Swab* Collection, by Randomized Group (HPV 16/18/31/33/45/52/58 mITT Cohort) ........ 5

Supplementary Table 4 | Number of Endpoint Swabs per Participant, by Randomized Group (HPV 16/18/31/33/45/52/58 mITT Cohort) ........ 6

Supplementary Table 5 | Incidence of persistent combined non-vaccine HPV types Pre- and Post-participant Crossover Vaccine Receipt, by group (non-vaccine type DNA negative subgroup of HVP 16/18 mITT cohort)........ 7

Supplementary Table 6 | Summary of mITT Endpoint HPV Types by Randomized Group Pre- and Post-participant Crossover (HPV 16/18 mITT Cohort) ........ 8

Supplementary Table 7 | Summary of HPV 16/18/31/33/45/52/58 mITT Endpoint HPV Types by Randomized Group Pre-and Post-participant Crossover (HPV 16/18/31/33/45/52/58 mITT Cohort) ........ 9

Supplementary Table 8 | Sensitivity Analysis - Post-Participant Crossover Incidence Excluding Participants with Evidence of Current or Past HPV Infection at Crossover or 3 Months post-Crossover, by Randomized Group ........ 10

Supplementary Table 9 | Baseline Characteristics of the ITT population ........ 11

Supplementary Table 10 | Sensitivity Analysis - Incidence of persistent HPV 16/18/31/33/45/52/58 infections and vaccine effectiveness within subgroups defined by enrollment cervical swab DNA positivity, by Arm (ITT cohort, Primary Endpoint Period)........ 12

Supplementary Table 11 | Sensitivity Analysis – Vaccine Efficacy as a Function of Time since Vaccination (mITT Sensitivity Cohorts*)........ 13

Supplementary Figures ........ 14

Supplementary Figure 1 | Pre- and Post-participant Crossover Incidence of Persistent HPV 16/18 and Background Non-vaccine Types........ 15

Supplementary Figure 2 | Pre- and Post-participant Crossover Incidence of Persistent HPV 16/18/31/33/45/52/58 and Background Non-vaccine Types ........ 16

Supplementary Figure 3 | Event Plot for Incident Persistent HPV 16/18 Infections (ITT cohort, Primary Endpoint Period, n=2,275)........ 17

Supplementary Figure 4 | Event Plot for Incident Persistent HPV 16/18/31/33/45/52/58 Infections (ITT cohort, Primary Endpoint Period, n=2,275) ........ 18

# Supplementary Information

## KEN SHE Study Team

**Bill and Melinda Gates Foundation** (Peter Dull, Christopher Gill, Reena Gulati, Abdul Rawuf Yousufzay, Sara Vernam); **Division of Infectious Diseases, Department of Medicine, Massachusetts General Hospital** (Ruanne Barnabas, Kate Heller, Meighan Krows, Diane Kanjilal, Edward Catalano, Odun Talabi), **Fred Hutchinson Cancer Center** (Elizabeth Brown, Denise Galloway, Jody Carter, Marci Wright, Priya R. Prabhu, Robin Smith); **KEMRI Kisumu** (Elizabeth A. Bukusi, Maricianah Onono, Imeldah N. Wakhungu, Annette A. Opondo, Catherine W. Mwakio, Christine A. Olweny, Cynthia Akinyi, David E. Muhoma, Debora A. Odhiambo, Donnavane A. Ondego, Florence A. Ondiek, George O. Omondi, Gilbert C. Mutai, Hellen A. Olweyo, Imelda N. Imali, Samya S. Rashid , Janet A. Okeyo, Irene Okumu, Joan A. Ongere, Job A. Ouma, Kevin O. Onyango, Linet A. Okode, Lizzie N. Kabete, Lyna A. Memo, Maqline A. Achola, Meldah O. Adipo, Mildred A. Owenga, Millicent A. Oronje, Moses O. Siaji, Nobert B. Walusala, Nollyne A. Okuku, Penina N. Amboka, Rebecca A. Otieno, Reina Lenturkana, Robai Mituyi, Simon M. Muthusi, Veronica O. Atogo, Dennis Kegode, Daisy Chepkoros, Ivy M. Mutuiri, Benard M. Muga, Caren A. Wemali, Enericah K. Kanampiu, Geoffrey Kebaso, Mildred Imbayi, Teresia O. Akinyi, Rebecca A. Otieno, Esther A. Odeny, Elijah Mbuya, Stephen O. Abiero, Roseline Sikolia, David N. Marwa, Peter O. Mboya, Elizabeth L. Musi, Beryl A. Osoga, Vincent R. Ochuka, Vincent O. Odera, Lydiah A. Okumu, Pius O. Atonga, Nollyne A. Okuku, Vincent K. Salano, Adero J. Cate, Nicholas Walukana, Timothy Kwena, Celestine Lihavi, Maureen A. Ochieng, Robai M. Mituyi, Perez O. Odhiambo, Oyamo O. Christopher, Kathrin L Amukonyi, Patricia Matti, Bill Nyongesa, Belder A. Odedo, Terrence Makani, Cynthia Otieno, Headmond O. Juma); **KEMRI Nairobi** (Betty Njoroge, Alice Njoki, Celina Muthii, Edna Nyandiga, Esther Neema, John Okumu, Ian Ng'ang'a, Paul Mutunga, Syovata Kimanthi, Umi W. Mugo, Victor Omondi, Vincent Juma); **KEMRI Thika** (Nelly Mugo, Agata Thumi, Anne Gaitho, Caren Koli, Catherine Kiptinness, Charlene Biwott, David Chege, Dorcas Kiboi, Edwin Mugo, Emily Anyango, Erick Koome, Faith Munyaka,Faith Rolex, Francis Khaemba, Fridah Nkatha, Gladys Namboka, Grace Ndung'u, Irene Kamau, Irene Njeru, Innes Wambui, Jacinta Nyokabi, Jane Gacheru, Jemimah Nyakio, John Njoroge,Joseph Gichuru, Josephine Njeri,Kelvin Mutugi, Kenneth Ngure, Linda Orwa, Linet Makena, Lynda Oluoch, Margaret Mwangi, Mary Kibatha, Mathew Irungu, Matilda Saina, Nina Ouko, Peter Mwenda, Peter Nzuve, Rispa Nduuru, Rose Odera,Sabina Ndichu, Sammy Ng'ang'a, Sarah Mbaire, Sarah Njoroge, Scholastica Wanjiku, Solomon Maina, Stanley Mwangi, Stephen Gakuo, Susan Njoroge,Veronica Muchoki, Victoria Wambui, Victor Munene, Vincent Juma, Virginia Wangechi, Zachary Gathu); **University of Washington, Mombasa** (R. Scott McClelland, Emmanuel Kabare, Fatma H. Mwidadi, Juma Shafi, Khamis Mwinyikai, Rukiya Hassan, Salwa Mustafa); **University of Washington, Seattle** (Elizabeth Brown, Connie Celum, Elena A. Rechkina, Jared M. Baeten, John Lin, Rachel Johnson, Rachel L. Winer, Stephen L. Cherne, Susan Morrison); **DF/Net Research, Inc., Seattle** (Angela Williams, Amra Hercinovic, Samantha Soonderjee, Gavin Robertson, Lisa Ondrejcek).

# Supplementary Tables

## Supplementary Table 1 | Completeness of Endpoint Swab* Collection, by Randomized Group (HPV 16/18 mITT Cohort)

| Pre-Crossover | | | | | | | | | | | | |
|---|---|---|---|---|---|---|---|---|---|---|---|---|
| | Swab 1 (Month 6) | | Swab 2 (Month 12) | | Swab 3 (Month 18) | | Swab 4 (Month 24) | | Swab 5 (Month 30 or Crossover) | | Swab 6 (Month 36 or Crossover) | |
| Randomized Group | Expected (n)** | Completed, n (%) | Expected (n)** | Completed, n (%) | Expected (n)** | Completed, n (%) | Expected (n)** | Completed, n (%) | Expected (n)** | Completed, n (%) | Expected (n)** | Completed, n (%) |
| Nonavalent HPV | 496 | 496 (100.0%) | 496 | 495 (99.8%) | 496 | 491 (99.0%) | 496 | 487 (98.2%) | 496 | 463 (93.3%) | 335 | 301 (89.9%) |
| Bivalent HPV | 489 | 489 (100.0%) | 489 | 487 (99.6%) | 489 | 484 (99.0%) | 489 | 479 (98.0%) | 489 | 465 (95.1%) | 325 | 299 (92.0%) |
| Control | 473 | 473 (100.0%) | 473 | 472 (99.8%) | 473 | 467 (98.7%) | 472 | 459 (97.2%) | 471 | 441 (93.6%) | 314 | 268 (85.4%) |
| All | 1458 | 1458 (100.0%) | 1458 | 1454 (99.7%) | 1458 | 1442 (98.9%) | 1457 | 1425 (97.8%) | 1456 | 1369 (94.0%) | 974 | 868 (89.1%) |

| Post-Crossover | | | | | | |
|---|---|---|---|---|---|---|
| | Swab 1 (Month 36 42 - Crossover M6) | | Swab 2 (Month 42 48 - Crossover M12) | | Swab 3 (Month 48 54 - Crossover M18) | |
| Randomized Group | Expected (n)** | Completed, n (%) | Expected (n)** | Completed, n (%) | Expected (n)** | Completed, n (%) |
| Nonavalent HPV | 476 | 472 (99.2%) | 476 | 467 (98.1%) | 476 | 438 (92.0%) |
| Bivalent HPV | 465 | 453 (97.4%) | 465 | 451 (97.0%) | 465 | 420 (90.3%) |
| Control | 449 | 444 (98.9%) | 449 | 433 (96.4%) | 449 | 405 (90.2%) |
| All | 1390 | 1369 (98.5%) | 1390 | 1351 (97.2%) | 1390 | 1263 (90.9%) |

*Endpoint swabs defined as post-Month 3 cervical or self-collected vaginal swabs at least 4 months apart.

**Pre-crossover swabs 1 through 5 are expected for all non-deceased participants. Pre-crossover swab 6 is expected for participants who either crossed over or exited at month 36. Post-crossover swabs 1 through 3 are expected for all participants who received crossover vaccine.

## Supplementary Table 2 | Number of Endpoint Swabs per Participant, by Randomized Group (HPV 16/18 mITT Cohort)

| Pre-Crossover: Number of Endpoint Swabs per Participant* | | | | | | | | | | | | | | |
|---|---|---|---|---|---|---|---|---|---|---|---|---|---|---|
| | 1 | | 2 | | 3 | | 4 | | 5 | | 6 | | Total | |
| **Randomized Group** | **n** | **Row %** | **n** | **Row %** | **n** | **Row %** | **n** | **Row %** | **n** | **Row %** | **n** | **Row %** | **n** | **%** |
| Nonavalent HPV | 1 | 0.1% | 4 | 0.4% | 4 | 0.4% | 24 | 2.4% | 162 | 16.3% | 301 | 30.3% | 496 | 34.0% |
| Bivalent HPV | 2 | 0.2% | 3 | 0.3% | 5 | 0.5% | 14 | 1.4% | 166 | 17.0% | 299 | 30.6% | 489 | 33.5% |
| Control | 1 | 0.1% | 5 | 0.5% | 8 | 0.8% | 18 | 1.9% | 173 | 18.3% | 268 | 28.3% | 473 | 32.4% |
| | 4 | 0.3% | 12 | 0.8% | 17 | 1.2% | 56 | 3.8% | 501 | 34.4% | 868 | 59.5% | 1458 | 100.0% |

| Post-Crossover: Number of Endpoint Swabs per Participant* | | | | | | | | | | |
|---|---|---|---|---|---|---|---|---|---|---|
| | 0 | | 1 | | 2 | | 3 | | Total | |
| **Randomized Group** | **n** | **Row %** | **n** | **Row %** | **n** | **Row %** | **n** | **Row %** | **n** | **%** |
| Nonavalent HPV | 4 | 0.8% | 5 | 1.1% | 29 | 6.1% | 438 | 92.0% | 476 | 34.2% |
| Bivalent HPV | 12 | 2.6% | 2 | 0.4% | 31 | 6.7% | 420 | 90.3% | 465 | 33.5% |
| Control | 5 | 1.1% | 11 | 2.4% | 28 | 6.2% | 405 | 90.2% | 449 | 32.3% |
| | 21 | | 18 | | 88 | | 1263 | | 1390 | 100.0% |

*Endpoint swabs defined as post-Month 3 cervical vaginal or self-collected vaginal swabs at least 4 months apart.

## Supplementary Table 3 | Completeness of Endpoint Swab* Collection, by Randomized Group (HPV 16/18/31/33/45/52/58 mITT Cohort)

| | Pre-Crossover | | | | | | | | | | | |
|---|---|---|---|---|---|---|---|---|---|---|---|---|
| | Swab 1 (Month 6) | | Swab 2 (Month 12) | | Swab 3 (Month 18) | | Swab 4 (Month 24) | | Swab 5 (Month 30 or Crossover) | | Swab 6 (Month 36 or Crossover) | |
| **Randomized Group** | **Expected (n)**** | **Completed, n (%)** | **Expected (n)**** | **Completed, n (%)** | **Expected (n)**** | **Completed, n (%)** | **Expected (n)**** | **Completed, n (%)** | **Expected (n)**** | **Completed, n (%)** | **Expected (n)**** | **Completed, n (%)** |
| Nonavalent HPV | 325 | 325 (100.0%) | 325 | 325 (100.0%) | 325 | 323 (99.4%) | 325 | 321 (98.8%) | 325 | 306 (94.2%) | 231 | 215 (93.1%) |
| Control | 290 | 290 (100.0%) | 290 | 289 (99.7%) | 290 | 287 (99.0%) | 290 | 282 (97.2%) | 290 | 273 (94.1%) | 195 | 167 (85.6%) |
| All | 615 | 615 (100.0%) | 615 | 614 (99.8%) | 615 | 610 (99.2%) | 615 | 603 (98.0%) | 615 | 579 (94.1%) | 426 | 382 (89.7%) |

| | Post-Crossover | | | | | |
|---|---|---|---|---|---|---|
| | Swab 1 (Month 36 42 - Crossover M6) | | Swab 2 (Month 42 48 - Crossover M12) | | Swab 3 (Month 48 54 - Crossover M18) | |
| **Randomized Group** | **Expected (n)**** | **Completed, n (%)** | **Expected (n)**** | **Completed, n (%)** | **Expected (n)**** | **Completed, n (%)** |
| Nonavalent HPV | 314 | 311 (99.0%) | 314 | 308 (98.1%) | 314 | 290 (92.4%) |
| Control | 279 | 275 (98.6%) | 279 | 267 (95.7%) | 279 | 250 (89.6%) |
| All | 593 | 586 (98.8%) | 593 | 575 (97.0%) | 593 | 540 (91.1%) |

*Endpoint swabs defined as post-Month 3 cervical or self-collected vaginal swabs at least 4 months apart.

**Pre-crossover swabs 1 through 5 are expected for all non-deceased participants. Pre-crossover swab 6 is expected for participants who either crossed over or exited at month 36. Post-crossover swabs 1 through 3 are expected for all participants who received crossover vaccine.

## Supplementary Table 4 | Number of Endpoint Swabs per Participant, by Randomized Group (HPV 16/18/31/33/45/52/58 mITT Cohort)

| Pre-Crossover: Number of Endpoint Swabs per Participant* | | | | | | | | | | | | | | |
|---|---|---|---|---|---|---|---|---|---|---|---|---|---|---|
| | 1 | | 2 | | 3 | | 4 | | 5 | | 6 | | Total | |
| **Randomized Group** | **n** | **Row %** | **n** | **Row %** | **n** | **Row %** | **n** | **Row %** | **n** | **Row %** | **n** | **Row %** | **n** | **%** |
| Nonavalent HPV | 0 | 0.0% | 2 | 0.3% | 2 | 0.3% | 15 | 2.3% | 91 | 14.0% | 215 | 33.1% | 325 | 52.8% |
| Control | 1 | 0.2% | 2 | 0.3% | 5 | 0.9% | 9 | 1.6% | 106 | 18.3% | 167 | 28.8% | 290 | 47.2% |
| | 1 | 0.2% | 4 | 0.7% | 7 | 1.1% | 24 | 3.9% | 197 | 32.0% | 382 | 62.1% | 615 | 100.0% |

| Post-Crossover: Number of Endpoint Swabs per Participant* | | | | | | | | | | |
|---|---|---|---|---|---|---|---|---|---|---|
| | 0 | | 1 | | 2 | | 3 | | Total | |
| **Randomized Group** | **n** | **Row %** | **n** | **Row %** | **n** | **Row %** | **n** | **Row %** | **n** | **%** |
| Nonavalent HPV | 3 | 1.0% | 3 | 1.0% | 18 | 5.7% | 290 | 92.4% | 314 | 53.0% |
| Control | 4 | 1.4% | 8 | 2.9% | 17 | 6.1% | 250 | 89.6% | 279 | 47.0% |
| | 7 | | 11 | | 35 | | 540 | | 593 | 100.0% |

*Endpoint swabs defined as post-Month 3 cervical vaginal or self-collected vaginal swabs at least 4 months apart.

## Supplementary Table 5 | Incidence of persistent combined non-vaccine HPV types Pre- and Post-participant Crossover Vaccine Receipt, by group (non-vaccine type DNA negative subgroup of HVP 16/18 mITT cohort)

**Pre-participant Crossover**

| Arm | HPV 16/18 naive at baseline (mITT) (n) | Non-vaccine HPV type DNA negative at baseline(n) | Number at risk (n) | Incident persistent non-vaccine HPV(n) | Woman-years of Follow-up† | Incidence of persistent non-vaccine HPV per 100 Woman-years | 95% Confidence Interval* Lower Bound | 95% Confidence Interval* Upper Bound | Incidence Rate Difference[β] (95% CI) | p-value[β] |
|---|---|---|---|---|---|---|---|---|---|---|
| Delayed HPV Vaccine | 473 | 203 | 203 | 97 | 417.56 | 23.23 | 18.84 | 28.34 | -2.56 (-8.39, 3.26) | 0.388 |
| Immediate HPV Vaccine | 985 | 397 | 397 | 203 | 786.98 | 25.79 | 22.37 | 29.60 | | |
| All | 1458 | 600 | 600 | 300 | 1204.53 | 24.91 | 22.17 | 27.89 | | |

**Post-participant Crossover**

| Arm | HPV 16/18 naive at baseline (mITT)(n) | Non-vaccine HPV type DNA negative at baseline (mITT) (n) | Number at risk (n) | Incident persistent non-vaccine HPV (n) | Woman-years of Follow-up ᵞ | Incidence of persistent non-vaccine HPV per 100 Woman-years | 95% Confidence Interval* Lower Bound | 95% Confidence Interval* Upper Bound | Incidence Rate Difference[β] (95% CI) | p-value[β] |
|---|---|---|---|---|---|---|---|---|---|---|
| Delayed HPV Vaccine | 473 | 203 | 98 | 23 | 113.98 | 20.18 | 12.79 | 30.28 | -2.47 (-12.9, 7.97) | 0.643 |
| Immediate HPV Vaccine | 985 | 397 | 182 | 48 | 211.94 | 22.65 | 16.70 | 30.03 | | |
| All | 1458 | 600 | 280 | 71 | 325.92 | 21.78 | 17.01 | 27.48 | | |

*Exact 95% confidence interval for incidence rate computed using the Poisson distribution.

† Follow-up time among participants HPV 16/18 naïve and non-vaccine HPV type negative at baseline, from enrollment to first positive result of consecutive positive HPV DNA results for participants with an endpoint, or from enrollment to last HPV DNA result not meeting endpoint criteria prior to crossover vaccine receipt for participants with no endpoint. For participants with an endpoint, result day is set to the mid-point between the first positive result defining the endpoint and the previous endpoint swab collection date for the calculation of follow-up time.

ᵞ Follow-up time among participants HPV 16/18 naïve and non-vaccine HPV type negative at baseline who received crossover vaccine and who were still at risk for incident non-vaccine type HPV (not previously censored and contributed at least one post-crossover swab), from date of crossover vaccine receipt to first positive result of consecutive positive HPV DNA results for participants with an endpoint, or from date of crossover vaccine receipt to last HPV DNA result not meeting endpoint criteria for participants with no endpoint. For participants with an endpoint, result day is set to the mid-point between the first positive result defining the endpoint and the previous endpoint swab collection date for the calculation of follow-up time.

[β]Incidence rate difference ($IR_{Delayed} - IR_{Immediate}$) computed using separate Poisson regression models for pre- and post-participant crossover, with an offset of log woman-years and randomized group as the only predictor. The difference in rates, 95% CIs, and two-sided p-values were obtained by estimating a nonlinear function of the Poisson model parameters on the mean scale. No adjustments for multiple comparisons were made.

NOTE: The HPV 16/18 mITT cohort is comprised of participants HPV 16/18 DNA negative at month 0 and month 3, and HPV 16/18 antibody negative at month 0. The non-vaccine HPV type DNA negative subgroup is comprised of participants additionally HPV type negative for HPV 26/35/39/40/42/43/44/51/53/54/56/59/61/66/68/69/70/73/82 at month 0 and month 3. One participant in the HPV 16/18 mITT cohort is excluded from this analysis because the Luminex back up assay does not include HPV 43/44.

## Supplementary Table 6 | Summary of mITT Endpoint HPV Types by Randomized Group Pre- and Post-participant Crossover (HPV 16/18 mITT Cohort)

| Pre-participant Crossover | | | | |
|---|---|---|---|---|
| | Randomized Group | | | |
| | Nonavalent HPV | Bivalent HPV | Control | Total |
| HPV Type | n | n | n | n |
| 16 | 1 | 2 | 60 | 63 |
| 16,18 | 0 | 0 | 1 | 1 |
| 18 | 0 | 1 | 28 | 29 |
| All | 1 | 3 | 89 | 93 |

| Post-participant Crossover | | | | |
|---|---|---|---|---|
| | Randomized Group | | | |
| | Nonavalent HPV | Bivalent HPV | Control | Total |
| HPV Type | n | n | n | n |
| 16 | 2 | 0 | 3 | 5 |
| 18 | 1 | 1 | 4 | 6 |
| All | 3 | 1 | 7 | 11 |

**Supplementary Table 7 | Summary of HPV 16/18/31/33/45/52/58 mITT Endpoint HPV Types by Randomized Group Pre-and Post-participant Crossover (HPV 16/18/31/33/45/52/58 mITT Cohort)**

| Pre-participant Crossover | | | |
|---|---|---|---|
| | Randomized Group | | |
| | Nonavalent HPV | Control | Total |
| **HPV Type** | **n** | **n** | **n** |
| 16 | 1 | 22 | 23 |
| 16,52 | 0 | 2 | 2 |
| 16,52,58 | 0 | 1 | 1 |
| 16,58 | 0 | 1 | 1 |
| 18 | 0 | 12 | 12 |
| 18,58 | 0 | 2 | 2 |
| 31 | 1 | 6 | 7 |
| 31,58 | 0 | 1 | 1 |
| 33 | 0 | 3 | 3 |
| 33,52 | 0 | 1 | 1 |
| 45 | 0 | 10 | 10 |
| 45,52 | 0 | 2 | 2 |
| 52 | 0 | 15 | 15 |
| 52,58 | 0 | 1 | 1 |
| 58 | 3 | 19 | 22 |
| | 5 | 98 | 103 |

| Post-participant Crossover | | | |
|---|---|---|---|
| | Randomized Group | | |
| | Nonavalent HPV | Control | Total |
| **HPV Type** | **n** | **n** | **n** |
| 16 | 2 | 2 | 4 |
| 18 | 1 | 1 | 2 |
| 33 | 0 | 2 | 2 |
| 45 | 1 | 0 | 1 |
| 52 | 1 | 3 | 4 |
| 58 | 0 | 1 | 1 |
| | 5 | 9 | 14 |

## Supplementary Table 8 | Sensitivity Analysis - Post-Participant Crossover Incidence Excluding Participants with Evidence of Current or Past HPV Infection at Crossover or 3 Months post-Crossover, by Randomized Group

### a. HPV 16/18

| | | | | | | 95% Confidence Interval* | |
|---|---|---|---|---|---|---|---|
| **Arm** | **Received Crossover Vaccine (n)** | **HPV 16/18 naive at baseline and crossover (n)** | **Incident persistent HPV 16/18 (n)** | **Woman-years of Follow-up**** | **Incidence of persistent HPV 16/18 per 100 Woman-years** | **Lower Bound** | **Upper Bound** |
| Nonavalent HPV | 710 | 449 | 2 | 601.82 | 0.33 | 0.04 | 1.20 |
| Bivalent HPV | 707 | 427 | 1 | 577.67 | 0.17 | 0.00 | 0.97 |
| Control | 707 | 322 | 0 | 426.15 | 0.00 | 0.00 | 0.87 |
| All | 2124 | 1198 | 3 | 1605.63 | 0.19 | 0.04 | 0.55 |

*Exact 95% confidence interval for incidence rate computed using the Poisson distribution.

**Follow-up time amongst women HPV 16/18 antibody-negative at month 0, DNA-negative at month 0, month 3, crossover, and crossover month 3, and who did not have a HPV 16/18 endpoint prior to crossover. For non-endpoint participants, follow-up time is computed from crossover month 3 to the date of the last negative test, and for endpoint participants follow-up time is computed from crossover month 3 to the mid-point between the first positive result of the endpoint and the previous result.

### b. HPV 16/18/31/33/45/52/58

| | | | | | | 95% Confidence Interval* | |
|---|---|---|---|---|---|---|---|
| **Arm** | **Received Crossover Vaccine (n)** | **HPV 16/18/31/33/45/52/58 naive at baseline and crossover (n)** | **Incident persistent HPV 16/18/31/33/45/52/58 (n)** | **Woman-years of Follow-up**** | **Incidence of persistent HPV 16/18/31/33/45/52/58 per 100 Woman-years** | **Lower Bound** | **Upper Bound** |
| Nonavalent HPV | 710 | 272 | 3 | 359.41 | 0.83 | 0.17 | 2.44 |
| Control | 707 | 142 | 1 | 183.61 | 0.54 | 0.01 | 3.03 |
| All | 1417 | 414 | 4 | 543.03 | 0.74 | 0.20 | 1.89 |

*Exact 95% confidence interval for incidence rate computed using the Poisson distribution.

**Follow-up time amongst women HPV 16/18/31/33/45/52/58 antibody-negative at month 0, DNA-negative at month 0, month 3, crossover, and crossover month 3, and who did not have a HPV 16/18/31/33/45/52/58 endpoint prior to crossover. For non-endpoint participants, follow-up time is computed from crossover month 3 to the date of the last negative test, and for endpoint participants follow-up time is computed from crossover month 3 to the mid-point between the first positive result of the endpoint and the previous result.

## Supplementary Table 9 | Baseline Characteristics of the ITT population

| | | Nonavalent HPV | Bivalent HPV | Control | All |
|---|---|---|---|---|---|
| **Characteristic** | **Category** | 758 | 760 | 757 | 2275 |
| Age group (years) | 15-17 | 453 (59.8%) | 424 (55.8%) | 424 (56.0%) | 1301 (57.2%) |
| | 18-20 | 305 (40.2%) | 336 (44.2%) | 333 (44.0%) | 974 (42.8%) |
| Age (years) | Median (IQR) | 17 (16, 18) | 17 (16, 19) | 17 (16, 19) | 17 (16, 19) |
| Marital status | Never married | 728 (96.0%) | 713 (93.8%) | 712 (94.1%) | 2153 (94.6%) |
| | Married | 25 (3.3%) | 39 (5.1%) | 32 (4.2%) | 96 (4.2%) |
| | Previously Married | 4 (0.5%) | 6 (0.8%) | 13 (1.7%) | 23 (1.0%) |
| | Other | 1 (0.1%) | 2 (0.3%) | 0 (0.0%) | 3 (0.1%) |
| Education (highest level) | No schooling | 1 (0.1%) | 3 (0.4%) | 3 (0.4%) | 7 (0.3%) |
| | Primary school, some or complete | 52 (6.9%) | 47 (6.2%) | 61 (8.1%) | 160 (7.0%) |
| | Secondary school, some or complete | 553 (73.0%) | 551 (72.5%) | 550 (72.7%) | 1654 (72.7%) |
| | Post-secondary school | 152 (20.1%) | 159 (20.9%) | 143 (18.9%) | 454 (20.0%) |
| Earns an income of her own | No | 665 (87.7%) | 653 (85.9%) | 656 (86.7%) | 1974 (86.8%) |
| | Yes | 93 (12.3%) | 107 (14.1%) | 101 (13.3%) | 301 (13.2%) |
| Has a current main or steady sexual partner | No | 209 (27.6%) | 222 (29.2%) | 211 (27.9%) | 642 (28.2%) |
| | Yes | 549 (72.4%) | 538 (70.8%) | 546 (72.1%) | 1633 (71.8%) |
| Age when first had vaginal intercourse (years) | <15 | 190 (25.1%) | 180 (23.7%) | 170 (22.5%) | 540 (23.7%) |
| | 15-17 | 397 (52.4%) | 415 (54.6%) | 445 (58.8%) | 1257 (55.3%) |
| | >=18 | 148 (19.5%) | 155 (20.4%) | 130 (17.2%) | 433 (19.0%) |
| | Don't remember | 23 (3.0%) | 10 (1.3%) | 12 (1.6%) | 45 (2.0%) |
| Number of lifetime sexual partners | 1 | 464 (61.2%) | 484 (63.7%) | 444 (58.7%) | 1392 (61.2%) |
| | 2 | 195 (25.7%) | 176 (23.2%) | 194 (25.6%) | 565 (24.8%) |
| | >=3 | 99 (13.1%) | 100 (13.2%) | 119 (15.7%) | 318 (14.0%) |
| Condom use with last vaginal sex | No | 239 (31.5%) | 244 (32.1%) | 233 (30.8%) | 716 (31.5%) |
| | Yes | 358 (47.2%) | 365 (48.0%) | 367 (48.5%) | 1090 (47.9%) |
| | No sex in past year | 161 (21.2%) | 151 (19.9%) | 157 (20.7%) | 469 (20.6%) |
| Syphilis | Negative | 757 (99.9%) | 760 (100.0%) | 754 (99.6%) | 2271 (99.8%) |
| | Positive | 1 (0.1%) | 0 | 1 (0.1%) | 2 (0.1%) |
| | Not Done | 0 | 0 | 2 (0.3%) | 2 (0.1%) |
| C. trachomatis | Negative | 665 (87.7%) | 663 (87.2%) | 651 (86.0%) | 1979 (87.0%) |
| | Positive | 93 (12.3%) | 97 (12.8%) | 106 (14.0%) | 296 (13.0%) |
| N. gonorrhoeae | Negative | 745 (98.3%) | 738 (97.1%) | 741 (97.9%) | 2224 (97.8%) |
| | Positive | 13 (1.7%) | 22 (2.9%) | 16 (2.1%) | 51 (2.2%) |
| HSV-2 | Negative | 616 (81.3%) | 597 (78.6%) | 584 (77.1%) | 1797 (79.0%) |
| | Positive | 141 (18.6%) | 162 (21.3%) | 173 (22.9%) | 476 (20.9%) |
| | Indeterminate | 1 (0.1%) | 1 (0.1%) | 0 | 2 (0.1%) |
| Bacterial vaginosis* | Negative | 604 (79.7%) | 576 (75.8%) | 587 (77.5%) | 1767 (77.7%) |
| | Positive | 154 (20.3%) | 183 (24.1%) | 170 (22.5%) | 507 (22.3%) |
| | Not Done | 0 | 1 (0.1%) | 0 | 1 (0.0%) |
| Trichomonas vaginalis | Negative | 723 (95.4%) | 728 (95.8%) | 722 (95.4%) | 2173 (95.5%) |
| | Positive | 35 (4.6%) | 32 (4.2%) | 35 (4.6%) | 102 (4.5%) |
| **HPV DNA**** | | | | | |
| HPV 16/18 | Positive | 112 (14.8%) | 124 (16.3%) | 131 (17.3%) | 367 (16.1%) |
| HPV 16/18/31/33/45/52/58 | Positive | 242 (31.9%) | 257 (33.8%) | 261 (34.5%) | 760 (33.4%) |
| **HPV Serum Antibodies (Luminex)** | | | | | |
| HPV 16/18 | Positive | 108 (14.3%) | 104 (13.7%) | 114 (15.1%) | 326 (14.3%) |
| HPV 16/18/31/33/45/52/58 | Positive | 212 (28.0%) | 212 (27.9%) | 221 (29.2%) | 645 (28.4%) |

*BV positive defined as Nugent Score 7-10..**Includes any DNA positive from enrollment cervical or external vaginal swabs.

**Supplementary Table 10 | Sensitivity Analysis - Incidence of persistent HPV 16/18/31/33/45/52/58 infections and vaccine effectiveness within subgroups defined by enrollment cervical swab DNA positivity, by Arm (ITT cohort, Primary Endpoint Period)**

| | | | | | | 95% Confidence Interval* | | Statistical Comparisons**** | | | |
|---|---|---|---|---|---|---|---|---|---|---|---|
| **Positive Cervical HPV 16/18/31/33/45/52/58 DNA at Baseline**** | **Arm** | **Enrolled (n)** | **Total Incident Persistent HPV 16/18/31/33/45/52/58 Infections (n)** | **Woman-years of Follow-up***** | **Incidence of persistent HPV 16/18/31/33/45/52/58 infections per 100 Woman-years** | **Lower Bound** | **Upper Bound** | **Comparison** | **Vaccine Effectiveness** | **95% CI** | **P-value** |
| No | Nonavalent HPV | 564 | 34 | 1400.15 | 2.43 | 1.68 | 3.39 | Nonavalent HPV v. Control | 87.40% | (82.0%, 91.2%) | <0.0001 |
| | Control | 539 | 259 | 1343.59 | 19.28 | 17.00 | 21.77 | | | | |
| | All | 1103 | 293 | 2743.74 | 10.68 | 9.49 | 11.97 | | | | |
| Yes | Nonavalent HPV | 194 | 16 | 473.61 | 3.38 | 1.93 | 5.49 | Nonavalent HPV v. Control | 80.61% | (67.0%, 88.6%) | <0.0001 |
| | Control | 218 | 93 | 533.81 | 17.42 | 14.06 | 21.34 | | | | |
| | All | 412 | 109 | 1007.42 | 10.82 | 8.88 | 13.05 | | | | |
| All | All | 1515 | 402 | 3751.16 | 10.72 | 9.70 | 11.82 | | | | |

*Exact 95% confidence interval for incidence rate computed using the Poisson distribution.

**The enrollment cervical swab was positive for HPV 16/18/31/33/45/52/58. Two consecutive negative results of the same HPV type that was positive at enrollment were required to be evaluable for an endpoint of that HPV type.

***Follow-up time among randomized participants, from enrollment to last endpoint swab.

****Incidence rate ratios with 95% confidence intervals are estimated within subgroups using two separate Poisson regression models with a two-way class variable for vaccine arm as the only covariate, an offset of log(woman-years), and robust standard errors. Vaccine effectiveness and 95% CI computed from the incidence rate ratio as [100*(1-Point Estimate)]. Standard errors from the Poisson models were used to generate Z-scores against the null hypothesis of IRR=1.0 and compute corresponding two-sided p-values.

**Supplementary Table 11 | Sensitivity Analysis – Vaccine Efficacy as a Function of Time since Vaccination (mITT Sensitivity Cohorts*)**

## a. HPV 16/18 mITT Sensitivity*

| | | Incident persistent HPV 16/18 | | |
|---|---|---|---|---|
| **Randomized Group** | **HPV 16/18 DNA negative at baseline (n)*** | **Pre-participant crossover (n)** | **Post-participant crossover (n)** | **Vaccine Efficacy at Month 54 (95% CI)†** |
| Nonavalent HPV | 569 | 1 | 3 | 99.3% (96.2%, 99.9%) |
| Bivalent HPV | 561 | 4 | 1 | |
| Control | 543 | 103 | 7 | |

## b. HPV 16/18/31/33/45/52/58 mITT Sensitivity*

| | | Incident persistent HPV 16/18/31/33/45/52/58 (n) | | |
|---|---|---|---|---|
| **Randomized Group** | **HPV 16/18/31/33/45/52/58 DNA negative at baseline (n)*** | **Pre-participant crossover (n)** | **Post-participant crossover (n)** | **Vaccine Efficacy at Month 54 (95% CI)†** |
| Nonavalent HPV | 437 | 8 | 5 | 99.6% (97.9%, 99.9%) |
| Control | 392 | 140 | 14 | |

*The mITT sensitivity cohorts include participants who were HPV DNA negative at enrollment and month 3 (enrollment serum HPV antibody results not used).

†Vaccine efficacy as a function of time since vaccination computed using a Cox regression model with time-varying covariates for HPV vaccine status and time since HPV vaccination (VE=100*(1-HR at 54 months).

## Supplementary Figures

**Supplementary Figure 1 | Pre- and Post-participant Crossover Incidence of Persistent HPV 16/18 and Background Non-vaccine Types**

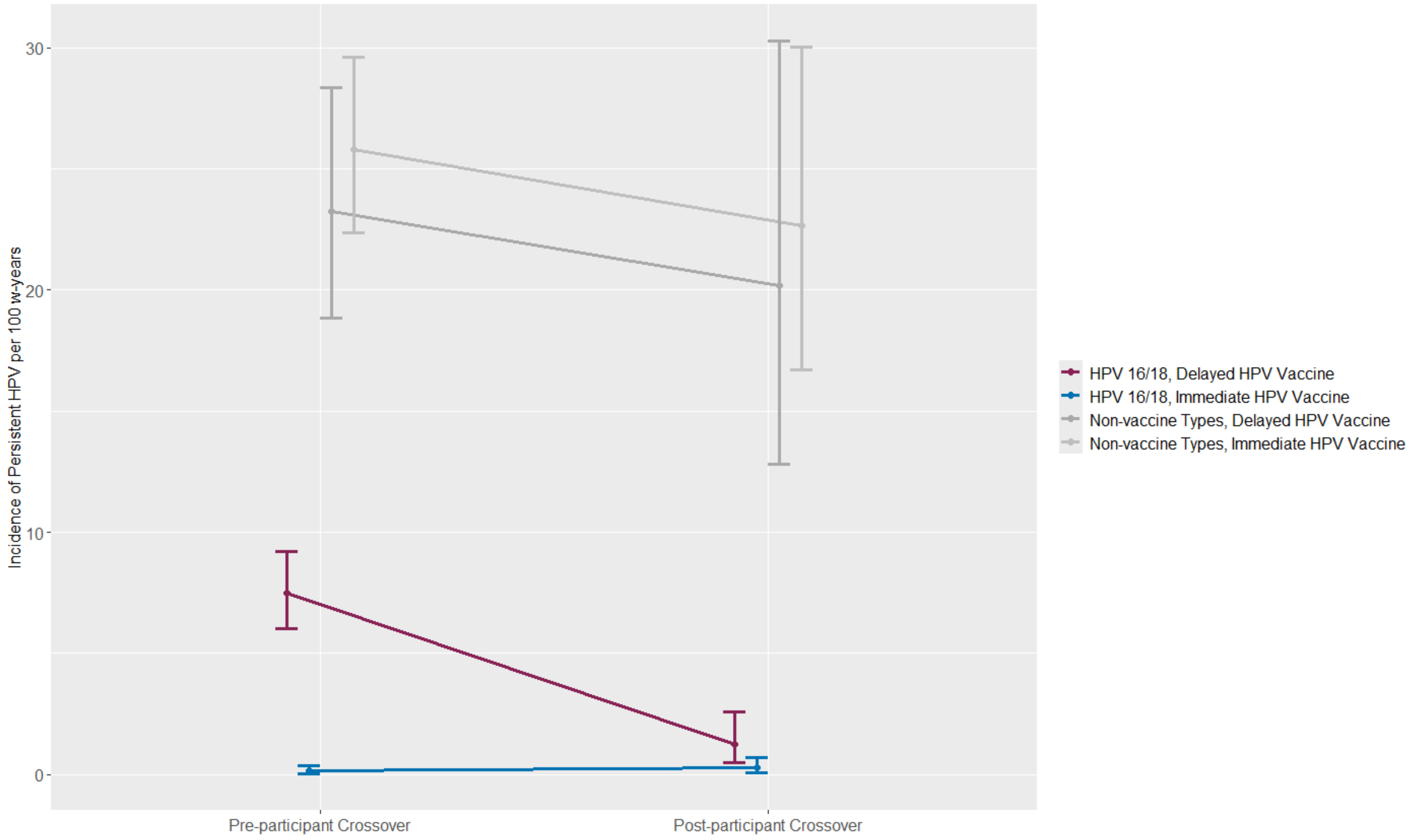


The blue and maroon lines represent the difference in pre- and post-participant crossover incidence of persistent HPV 16/18 in the HPV 16/18 modified intention-to-treat cohort (n=1,458 participants) for the immediate and delayed HPV vaccine groups, respectively. The grey lines represent the difference in incidence of non-vaccine type HPV 26/35/39/40/42/43/44/51/53/54/56/59/61/66/68/69/70/73/82 among HPV 16/18 mITT cohort participants who were also non-vaccine type negative at month 0 and month 3 (n=600 participants). Error bars represent the lower and upper bounds of the 95% confidence interval for each incidence rate, computed using the Poisson distribution.

**Supplementary Figure 2 | Pre- and Post-participant Crossover Incidence of Persistent HPV 16/18/31/33/45/52/58 and Background Non-vaccine Types**

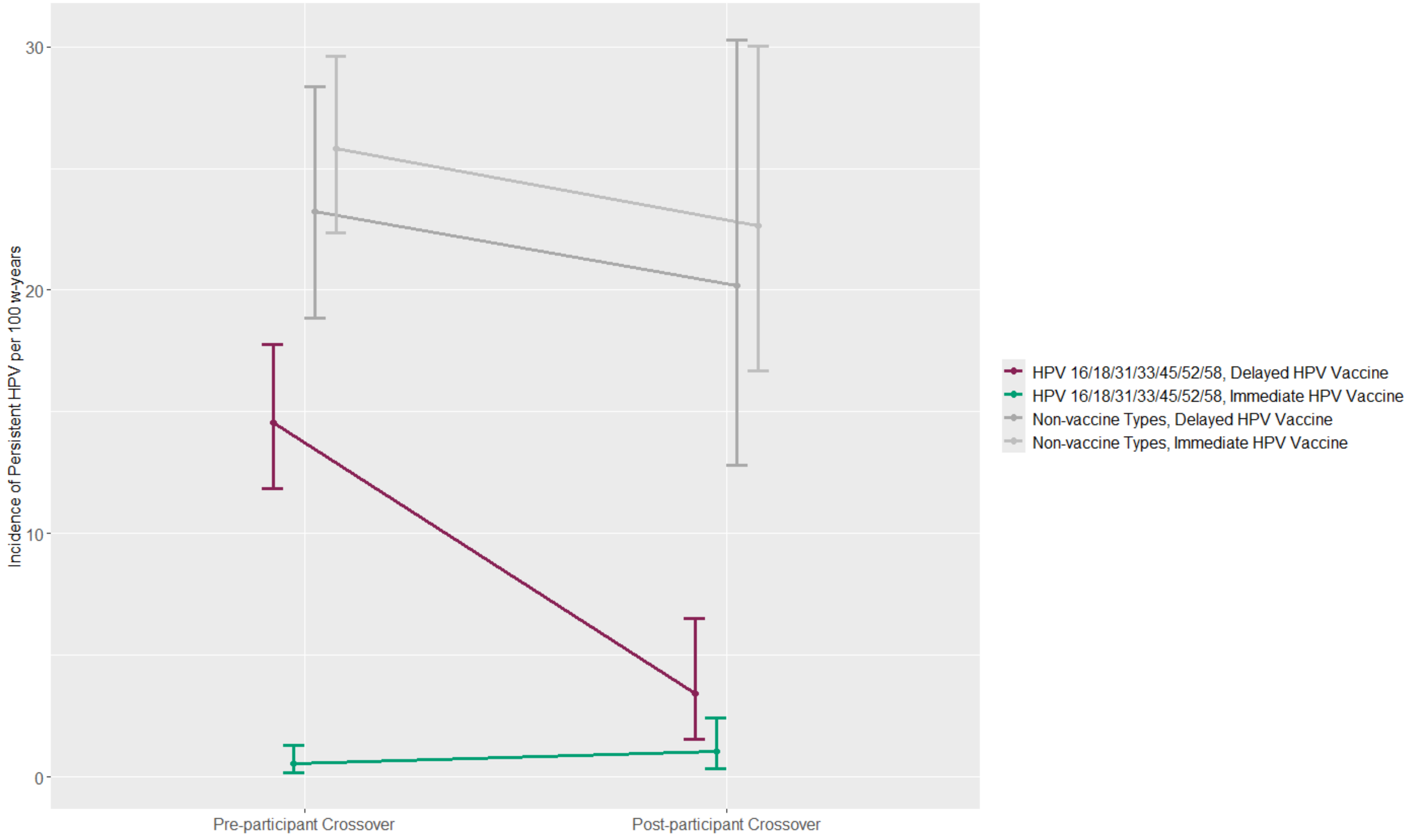


The green and maroon lines represent the difference in pre- and post-participant crossover incidence of persistent HPV 16/18/31/33/45/52/58 in the HPV 16/18/31/33/45/52/58 modified intention-to-treat cohort (n=615 participants) for the immediate and delayed HPV vaccine groups, respectively. The grey lines represent the difference in incidence of non-vaccine type HPV 26/35/39/40/42/43/44/51/53/54/56/59/61/66/68/69/70/73/82 among HPV 16/18 mITT cohort participants who were also non-vaccine type negative at month 0 and month 3 (n=600 participants). Error bars represent the lower and upper bounds of the exact 95% confidence interval for each incidence rate, computed using the Poisson distribution.

**Supplementary Figure 3 | Event Plot for Incident Persistent HPV 16/18 Infections (ITT cohort, Primary Endpoint Period, n=2,275)**

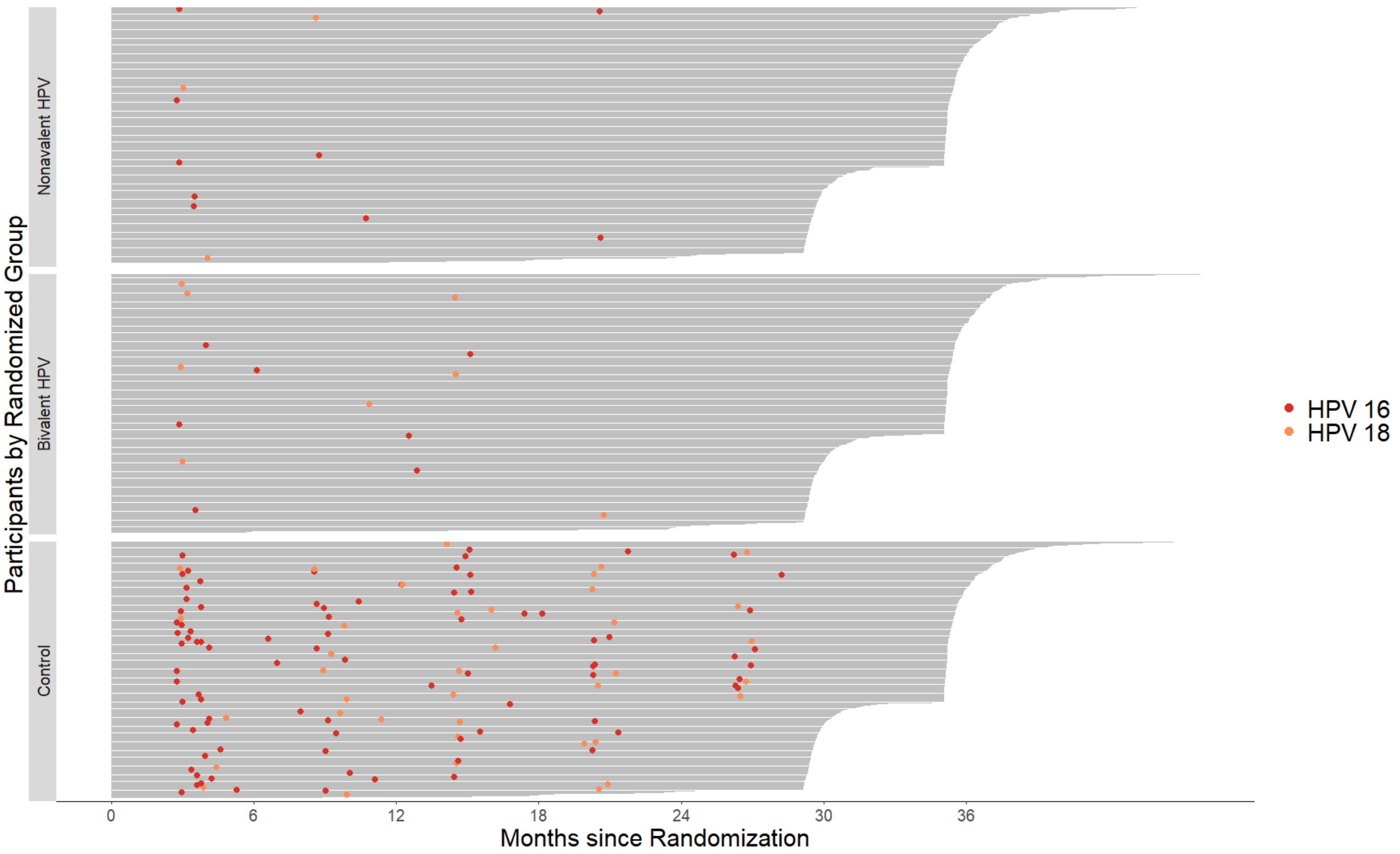


Recurrent event plot for incident persistent HPV 16 and HPV 18 in the intention-to-treat cohort (n=2,275 participants). The grey shaded areas represent individual participant follow-up time from randomization to the last endpoint swab at or prior to the crossover visit, and the dots represent HPV 16 and HPV 18 incident persistent type-specific infections for each participant plotted at months since randomization to the first positive swab of the incident persistent event. Participants with persistent infection at enrollment and/or during follow-up remained at risk for other HPV type infections. Two consecutive HPV negative results of the previously persistent positive HPV type were required to meet the definition of HPV clearance, at which time participants were eligible to contribute new incident persistent infections of that HPV type.

**Supplementary Figure 4 | Event Plot for Incident Persistent HPV 16/18/31/33/45/52/58 Infections (ITT cohort, Primary Endpoint Period, n=2,275)**

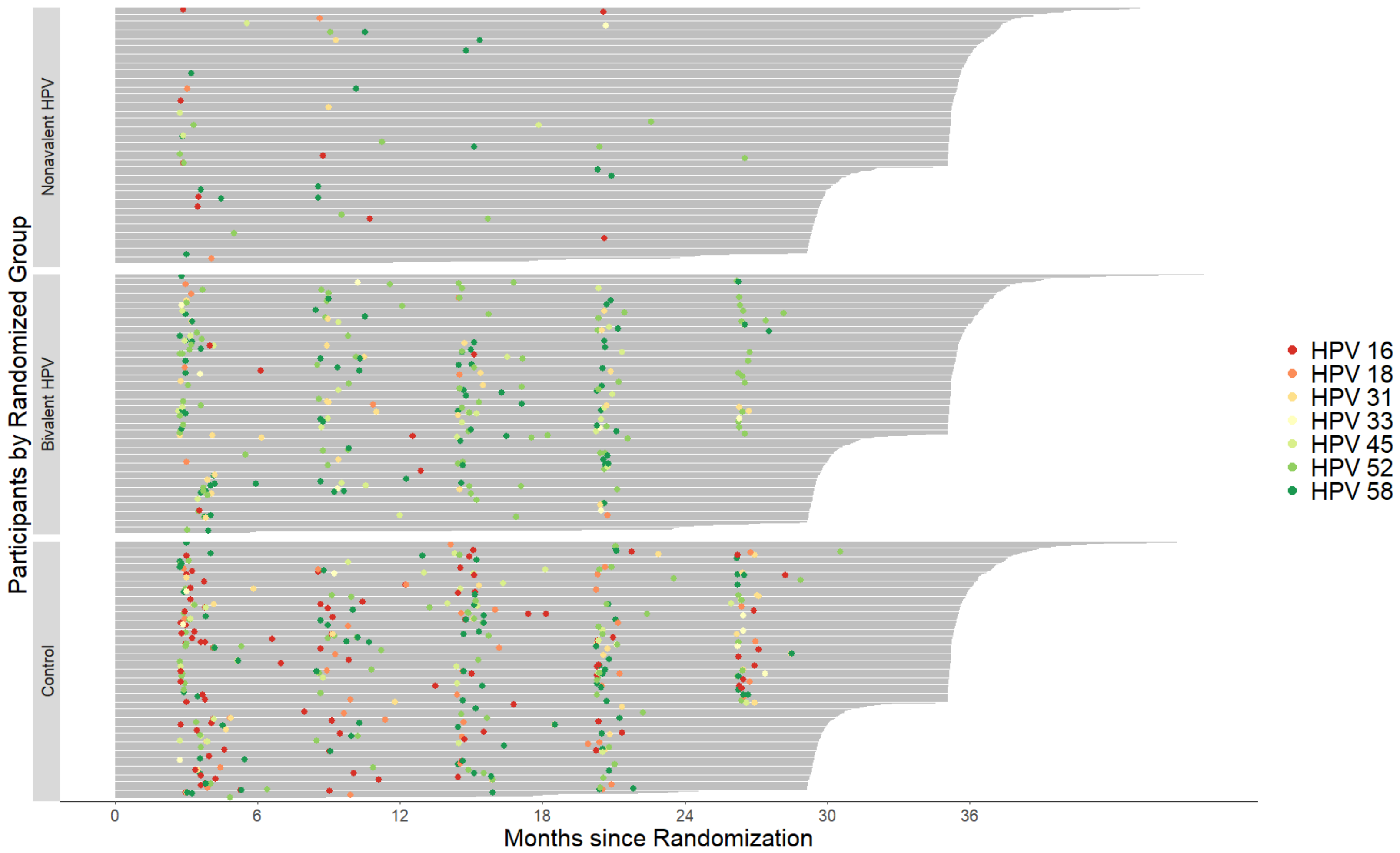


Recurrent event plot for incident persistent HPV 16, 18, 31, 33, 45, 52, and 58 in the intention-to-treat cohort (n=2,275 participants). The grey shaded areas represent individual participant follow-up time from randomization to the last endpoint swab at or prior to the crossover visit, and the dots represent HPV 16, 18, 31, 33, 45, 52, and 58 incident persistent type-specific infections for each participant plotted at months since randomization to the first positive swab of the incident persistent event. Participants with persistent infection at enrollment and/or during follow-up remained at risk for other HPV type infections. Two consecutive HPV negative results of the previously persistent positive HPV type were required to meet the definition of HPV clearance, at which time participants were eligible to contribute new incident persistent infections of that HPV type.

# CONSORT 2010 checklist of information to include when reporting a randomised trial*

| Section/Topic | Item No | Checklist item | Reported on page No |
|---|---|---|---|
| **Title and abstract** | | | |
| | 1a | Identification as a randomised trial in the title | 1 |
| | 1b | Structured summary of trial design, methods, results, and conclusions (for specific guidance see CONSORT for abstracts) | 2 |
| **Introduction** | | | |
| Background and objectives | 2a | Scientific background and explanation of rationale | 3-5 |
| | 2b | Specific objectives or hypotheses | 4-5 |
| **Methods** | | | |
| Trial design | 3a | Description of trial design (such as parallel, factorial) including allocation ratio | 15-16 |
| | 3b | Important changes to methods after trial commencement (such as eligibility criteria), with reasons | 15 |
| Participants | 4a | Eligibility criteria for participants | 16 |
| | 4b | Settings and locations where the data were collected | 15 |
| Interventions | 5 | The interventions for each group with sufficient details to allow replication, including how and when they were actually administered | 16-17 |
| Outcomes | 6a | Completely defined pre-specified primary and secondary outcome measures, including how and when they were assessed | 18-19 |
| | 6b | Any changes to trial outcomes after the trial commenced, with reasons | N/A |
| Sample size | 7a | How sample size was determined | N/A |
| | 7b | When applicable, explanation of any interim analyses and stopping guidelines | N/A |
| Randomisation: | | | |
| Sequence generation | 8a | Method used to generate the random allocation sequence | 16 |
| | 8b | Type of randomisation; details of any restriction (such as blocking and block size) | 16 |
| Allocation concealment mechanism | 9 | Mechanism used to implement the random allocation sequence (such as sequentially numbered containers), describing any steps taken to conceal the sequence until interventions were assigned | 16-17 |
| Implementation | 10 | Who generated the random allocation sequence, who enrolled participants, and who assigned participants to interventions | 16-17 |
| Blinding | 11a | If done, who was blinded after assignment to interventions (for example, participants, care providers, those | 16-17 |

| | | | |
|---|---|---|---|
| | | assessing outcomes) and how | |
| | 11b | If relevant, description of the similarity of interventions | 16-17 |
| Statistical methods | 12a | Statistical methods used to compare groups for primary and secondary outcomes | 19-22 |
| | 12b | Methods for additional analyses, such as subgroup analyses and adjusted analyses | 19-22 |
| **Results** | | | |
| Participant flow (a diagram is strongly recommended) | 13a | For each group, the numbers of participants who were randomly assigned, received intended treatment, and were analysed for the primary outcome | 5, Fig. 1 |
| | 13b | For each group, losses and exclusions after randomisation, together with reasons | 5, Fig. 1 |
| Recruitment | 14a | Dates defining the periods of recruitment and follow-up | 5 |
| | 14b | Why the trial ended or was stopped | n/a |
| Baseline data | 15 | A table showing baseline demographic and clinical characteristics for each group | 29-30 |
| Numbers analysed | 16 | For each group, number of participants (denominator) included in each analysis and whether the analysis was by original assigned groups | Fig. 1 |
| Outcomes and estimation | 17a | For each primary and secondary outcome, results for each group, and the estimated effect size and its precision (such as 95% confidence interval) | 6-10 |
| | 17b | For binary outcomes, presentation of both absolute and relative effect sizes is recommended | n/a |
| Ancillary analyses | 18 | Results of any other analyses performed, including subgroup analyses and adjusted analyses, distinguishing pre-specified from exploratory | 8-10 |
| Harms | 19 | All important harms or unintended effects in each group (for specific guidance see CONSORT for harms) | 10, 32 |
| **Discussion** | | | |
| Limitations | 20 | Trial limitations, addressing sources of potential bias, imprecision, and, if relevant, multiplicity of analyses | 12 |
| Generalisability | 21 | Generalisability (external validity, applicability) of the trial findings | 11-13 |
| Interpretation | 22 | Interpretation consistent with results, balancing benefits and harms, and considering other relevant evidence | 11-13 |
| **Other information** | | | |
| Registration | 23 | Registration number and name of trial registry | 2, 15 |
| Protocol | 24 | Where the full trial protocol can be accessed, if available | Suppl. Inf. 19 |
| Funding | 25 | Sources of funding and other support (such as supply of drugs), role of funders | 3, 26 |

Citation: Schulz KF, Altman DG, Moher D, for the CONSORT Group. CONSORT 2010 Statement: updated guidelines for reporting parallel group randomised trials. BMC Medicine. 2010;8:18.
© 2010 Schulz et al. This is an Open Access article distributed under the terms of the Creative Commons Attribution License (http://creativecommons.org/licenses/by/2.0), which permits unrestricted use, distribution, and reproduction in any medium, provided the original work is properly cited.

*We strongly recommend reading this statement in conjunction with the CONSORT 2010 Explanation and Elaboration for important clarifications on all the items. If relevant, we also recommend reading CONSORT extensions for cluster randomised trials, non-inferiority and equivalence trials, non-pharmacological treatments, herbal interventions, and pragmatic trials. Additional extensions are forthcoming: for those and for up-to-date references relevant to this checklist, see www.consort-statement.org.
