# Supplementary material for: Efficacy and durability of immediate versus delayed single-dose HPV vaccination for persistent infection among young women in Kenya: a randomized, blinded, cross-over clinical trial
Source: Nat Commun. 2026 May 11;17:6289. doi: 10.1038/s41467-026-72654-8 (PMC13377066; doi:10.1038/s41467-026-72654-8)
Supplement: Supplementary file 2 — Reporting Summary [file 41467_2026_72654_MOESM2_ESM.pdf]

Corresponding author(s): Ruanne V. Barnabas
Last updated by author(s): Apr 14, 2026

# Reporting Summary

Nature Portfolio wishes to improve the reproducibility of the work that we publish. This form provides structure for consistency and transparency in reporting. For further information on Nature Portfolio policies, see our Editorial Policies and the Editorial Policy Checklist.

## Statistics

For all statistical analyses, confirm that the following items are present in the figure legend, table legend, main text, or Methods section.

| n/a | Confirmed | |
|---|---|---|
| | x | The exact sample size (*n*) for each experimental group/condition, given as a discrete number and unit of measurement |
| | x | A statement on whether measurements were taken from distinct samples or whether the same sample was measured repeatedly |
| | x | The statistical test(s) used AND whether they are one- or two-sided<br>*Only common tests should be described solely by name; describe more complex techniques in the Methods section.* |
| | x | A description of all covariates tested |
| | x | A description of any assumptions or corrections, such as tests of normality and adjustment for multiple comparisons |
| | x | A full description of the statistical parameters including central tendency (e.g. means) or other basic estimates (e.g. regression coefficient) AND variation (e.g. standard deviation) or associated estimates of uncertainty (e.g. confidence intervals) |
| | x | For null hypothesis testing, the test statistic (e.g. *F*, *t*, *r*) with confidence intervals, effect sizes, degrees of freedom and *P* value noted<br>*Give P values as exact values whenever suitable.* |
| x | | For Bayesian analysis, information on the choice of priors and Markov chain Monte Carlo settings |
| x | | For hierarchical and complex designs, identification of the appropriate level for tests and full reporting of outcomes |
| | x | Estimates of effect sizes (e.g. Cohen's *d*, Pearson's *r*), indicating how they were calculated |

*Our web collection on statistics for biologists contains articles on many of the points above.*

## Software and code

Policy information about availability of computer code

| | |
|---|---|
| Data collection | Electronic case report forms (eCRFs) in DFExplore (DF/Net Research, Inc. ©, Seattle, WA, US) |
| Data analysis | SAS software, version 9.4 (SAS Institute, North Carolina, US) and R (version 4.2.2) |

For manuscripts utilizing custom algorithms or software that are central to the research but not yet described in published literature, software must be made available to editors and reviewers. We strongly encourage code deposition in a community repository (e.g. GitHub). See the Nature Portfolio guidelines for submitting code & software for further information.

## Data

Policy information about availability of data

All manuscripts must include a data availability statement. This statement should provide the following information, where applicable:

- Accession codes, unique identifiers, or web links for publicly available datasets
- A description of any restrictions on data availability
- For clinical datasets or third party data, please ensure that the statement adheres to our policy

Due to restrictions set forth by the KEMRI Scientific Ethics Review Unit (SERU), the KEN SHE study data cannot be made publicly available without SERU approval for its use. We have included the software in the manuscript and completed the Code Availability statement in the manuscript.

## Research involving human participants, their data, or biological material

Policy information about studies with human participants or human data. See also policy information about sex, gender (identity/presentation), and sexual orientation and race, ethnicity and racism.

| | |
|---|---|
| Reporting on sex and gender | Self-reported female sex at birth was an inclusion criterion for KEN SHE study enrollment. The KEN SHE study findings pertain to individuals with female sex at birth only. |
| Reporting on race, ethnicity, or other socially relevant groupings | The KEN SHE study did not collect data on race, ethnicity, or other social groupings. Minimal self-reported socioeconomic data (highest level of schooling and whether a participant earns an income on her own) was collected and previously reported. |
| Population characteristics | KEN SHE participants are young women, age 15-20, living in proximity to one of the three KEN SHE study sites in Thika, Nairobi, or Kisumu, Kenya. At the time of enrollment, participants had 1 to 5 lifetime sexual partners (inclusive), no history of previous HPV vaccination, and negative pregnancy and HIV rapid test results. |
| Recruitment | Participants were recruited through community outreach programs, which limits bias associated with facility-based recruitment but this is not a formal general population sample. |
| Ethics oversight | The Kenya Medical Research Institute (KEMRI) Scientific Ethics Review Unit (SERU) and Massachusetts General Hospital Institutional Review Board (MGH IRB) reviewed and approved the study protocol. |

Note that full information on the approval of the study protocol must also be provided in the manuscript.

# Field-specific reporting

Please select the one below that is the best fit for your research. If you are not sure, read the appropriate sections before making your selection.

☒ Life sciences ☐ Behavioural & social sciences ☐ Ecological, evolutionary & environmental sciences

For a reference copy of the document with all sections, see nature.com/documents/nr-reporting-summary-flat.pdf

# Life sciences study design

All studies must disclose on these points even when the disclosure is negative.

| | |
|---|---|
| Sample size | The sample size calculations also assumed a combined persistent HPV 16/18/31/33/45/52/58 annual incidence of 5%, single-dose vaccine efficacy of 75%, and loss-to-follow-up of 10% with a fixed follow-up time of 12 months. Sample size calculations assumed that 52% of participants would meet the requirements for inclusion in the primary analysis based on the observed prevalence of HPV infection in similar settings. Assuming a proportional hazards model (seqDesign in R) with 80% power to detect 75% efficacy, a sample size of 2,250 participants was planned. |
| Data exclusions | As pre-established in the statistical analysis plan, the primary analysis is conducted within the mITT cohorts for HPV 16/18 and HPV 16/18/31/33/45/52/58, which respectively exclude participants DNA- or antibody-positive at enrollment, or DNA-positive at month 3 for the HPV DNA types 16/18 or 16/18/31/33/45/52/58. The ITT analysis was conducted among all participants. |
| Replication | Analysis cohorts (n participants), vaccine efficacy (1-HR), vaccine effectiveness (1-IRR) and corresponding confidence intervals and p-values were coded independently by two analysts. All findings were replicated. |
| Randomization | An unblinded statistical analyst generated the randomization sequence using SAS v9.4. Randomization was stratified by site, using a fixed block size of 15 and a 1:1:1 allocation. Blinded study assignment was implemented via http://www.randomize.net (Ottawa, ON, Canada). |
| Blinding | Study staff, participants, investigators, clinic staff, lab technicians, the endpoints adjudication committee members, and other study team members did not have access to the randomization codes, except for the unblinded statistical analysts and unblinded pharmacists at each site. An unblinded pharmacist entered the participant identification number (PTID) on randomize.net, obtained the next sequential intervention assignment, recorded the PTID and randomization identifier on an eCRF, drew up the vaccine in a masked syringe, and administered the vaccination via the intramuscular route. An independent observer, not on the study team, observed the masked vaccination to assess the success of masking. Crossover vaccination was administered in a blinded manner. |

# Reporting for specific materials, systems and methods

We require information from authors about some types of materials, experimental systems and methods used in many studies. Here, indicate whether each material, system or method listed is relevant to your study. If you are not sure if a list item applies to your research, read the appropriate section before selecting a response.

## Materials & experimental systems

| n/a | Involved in the study |
| --- | --- |
| ☒ | ☐ Antibodies |
| ☒ | ☐ Eukaryotic cell lines |
| ☒ | ☐ Palaeontology and archaeology |
| ☒ | ☐ Animals and other organisms |
| ☐ | ☒ Clinical data |
| ☒ | ☐ Dual use research of concern |
| ☒ | ☐ Plants |

## Methods

| n/a | Involved in the study |
| --- | --- |
| ☒ | ☐ ChIP-seq |
| ☒ | ☐ Flow cytometry |
| ☒ | ☐ MRI-based neuroimaging |

# Clinical data

Policy information about clinical studies

All manuscripts should comply with the ICMJE guidelines for publication of clinical research and a completed CONSORT checklist must be included with all submissions.

Clinical trial registration: NCT03675256

Study protocol: The trial protocol paper has been published and the full protocol is available upon request lnakatsuka@partners.org.

Data collection: Study recruitment and enrollment occurred between December 20, 2018 and November 15, 2019, at three study sites in Thika, Nairobi, and Kisumu, Kenya.  Data collected between December 20, 2018 and June 14, 2024 are reported in this manuscript. The study was conducted at three Kenya Medical Research Institute (KEMRI) clinical sites in Kisumu, Thika, and Nairobi.

Outcomes: Pre-planned Primary Outcomes: Incident persistent HPV infection by vaccine type (HPV 16/18 for bivalent and nonavalent; HPV 16/18/31/33/45/52/58 for nonavalent), defined as detection at two consecutive visits at least four months apart. The primary objective evaluates vaccine efficacy (VE) durability for HPV 16/18 and HPV 16/18/31/33/45/52/58.
Pre-planned Exploratory Outcomes: Vaccine effectiveness in the ITT cohort (including those with prevalent infection at baseline); effectiveness among individuals with cervical infection at vaccination.

# Plants

Seed stocks: *Report on the source of all seed stocks or other plant material used. If applicable, state the seed stock centre and catalogue number. If plant specimens were collected from the field, describe the collection location, date and sampling procedures.*

Novel plant genotypes: *Describe the methods by which all novel plant genotypes were produced. This includes those generated by transgenic approaches, gene editing, chemical/radiation-based mutagenesis and hybridization. For transgenic lines, describe the transformation method, the number of independent lines analyzed and the generation upon which experiments were performed. For gene-edited lines, describe the editor used, the endogenous sequence targeted for editing, the targeting guide RNA sequence (if applicable) and how the editor was applied.*

Authentication: *Describe any authentication procedures for each seed stock used or novel genotype generated. Describe any experiments used to assess the effect of a mutation and, where applicable, how potential secondary effects (e.g. second site T-DNA insertions, mosiacism, off-target gene editing) were examined.*
