# Supplementary material for: Efficacy and durability of immediate versus delayed single-dose HPV vaccination for persistent infection among young women in Kenya: a randomized, blinded, cross-over clinical trial
Source: Nat Commun. 2026 May 11;17:6289. doi: 10.1038/s41467-026-72654-8 (PMC13377066; doi:10.1038/s41467-026-72654-8)
Supplement: Supplementary file 3 — Transparent Peer Review file [file 41467_2026_72654_MOESM3_ESM.pdf]

nature portfolio

# Peer Review File

# Efficacy and durability of immediate single-dose human papillomavirus vaccination versus delayed vaccination in persistent HPV infection among young women: a randomized, blinded, cross-over clinical trial

Corresponding Author: Professor Ruanne Barnabas

**This file contains all reviewer reports in order by version, followed by all author rebuttals in order by version.**

Version 0:

Reviewer comments:

Reviewer #1

(Remarks to the Author)
Very clear data on long-term efficacy of one-dose HPV vaccination among adolescent girls. Could have large public health impact on effective strategies for cervical cancer elimination.

The manuscript is very clear and carefully analysed. Comments:
It is not clear from what source population the participants derive. We are told inclusion and exclusion criteria and enrollment sites. Are they healthy volunteers sourced using advertisements, attending the hospitals for other reasons or enrolled some other way?

The impact of the baseline serologies is not clear. As policies are unlikely to use HPV serology at enrollment, a sensitivity analysis ignoring the serology analyses would have been helpful.

Most of the estimates in the text are presented with confidence intervals, but not all. It is not useful to state that the incidence is 0.00 if there is no confidence interval or at least a p-value. If the numbers are too small to do the statistics, please just delete these sentences.

Finally, I do not understand the sentence “upper age at vaccination at which point VE is presumed to have a small effect on dysplastic lesions and cancer”. The evidence fromRCTs in mid-adult women clearly show high VE in the mITT population (see the Cochrane reviews on this).

(Remarks on code availability)

Reviewer #2

(Remarks to the Author)
The MS is very well written,valid methodology eventhough there were no control as it is not feasible. The study results cover most of the objectives especially on the durability of the single dose at 54 months.However, there some points might consider to be added in the text and especially discuss in the discussion:
1. Data on Table a and b It is noted that the non vaccine types during the cross over vaccination period were much higher than during the enrollment vaccination period in all groups including the control group. Please discuss on this and its effects on the VE and implementation.
2. Similar pattern observed for the gonorhoea infections.
3. From the Figure 2a and 2b somehow, there is no decline or slightly decine in the cumulative incidence of HPV persistent infections after the delayed HPV vaccination. Please discuss on this and its effects if any as the delayed was about 30-36 motnhs.

(Remarks on code availability)

Reviewer #3

(Remarks to the Author)
The authors presented the findings for a clinical trial of the single-dose HPV vaccination. The findings provide evidence-based support for the efficacy of a single-dose schedule. This would help improve access to HPV information and, therefore, help accelerate the time of achieving the goal of cervical cancer elimination, particularly in resource-limited countries.
To help readers understand the content, more details could be provided.

A. Abstract
A.1. The time point at which the crossover was carried out should be included in the Abstract.
B.2. Whether the assessment at 54 months was pre-specified in the protocol? If yes, please state this. If not, please justify.

Main text
B. Title
B.1. Please include the age range and the targeted vaccinees (female or both sexes) in the title.

C. Introduction.
C.1. The introduction could be shortened, given that the current manuscript has exceeded the suggested word counts of the journal.
C.2. Please define "VE" when the term is used in the first
C.3. The first paragraph of page 5. "In 2023, only 27% of adolescent girls and young women globally were vaccinated against HPV." The authors should report vaccine uptake among low HDI countries. Or, please state clearly if "27%" refers to the uptake among low HDI countries globally.
Also, what is the age range of "young women" in the statement? Presumably, vaccine uptake decreases as age increases.
C.4. The first paragraph of page 5. "… 11.6 million cases of cervical cancer over their lifetimes". Please state the methodology for getting this number/estimate.
C.5. The second paragraph of page 5. "Single-dose HPV vaccination has high efficacy over three to five years. " Please add reference(s) for this statement, and state the condition for the findings. If the single-dose vaccination schedule has been shown/reported for having a high efficacy over 3-5 years, please include the added value for the current study that evaluated the efficacy at 54 months, i.e., 4.5 years, which is within the range of 3-5 years.

D. Study objective.
D.1. What is the maximum age of the study? It is unclear to state "15 years an older".
D.2. State the rationale for evaluating the efficacy at 54 months, i.e., 4.5 years, instead of a time point of an integer in years, in addition to referring readers to the protocol. Also, the author mentioned that this was "based on sustained antibody levels observed over 16 years". What is the connection between 54 months and 16 years?

E. Methods.
E.1. Please refer the readers to the flow chart when describing the vaccination schedules. The study design is unclear from the text only.
E.2. Study design. The paragraph "In GAVI-funded countries, …" is more related to the rationale of the study, rather than the methods. Consider moving the paragraph to a better position.
E.3. Cite references for the assessment of durability "If the vaccine is not durable (i.e., the vaccine effect wanes), we expect the coefficient for time since vaccination to be positive …" (page 12).
E.4. For the assessment of durability, the modified intention-to-treat (mITT) cohort/approach was considered. For the assessment of vaccine effectiveness, the intention-to-treat (ITT) cohort/approach was considered. The authors should explain the difference.

F. Results.
F.1. Second paragraph, page 15. "The incidence of persistent non-vaccine HPV types was comparable …" Please indicate the p-value of the comparison of the incidence between groups.
F.2. Please indicate the findings of "negative coefficients of time since vaccination" in the Results section.

G. Discussion
G.1. It would be interesting to discuss the rationale of the requirement of receiving three doses when HPV vaccines (both bivalent and quadrivalent by different manufacturers) were first available in the market.
G.2. When stating the limitations, the authors mentioned that "observational data for single-dose HPV vaccination support efficacy over a decade … We are collecting additional data in this cohort to 90 months post-vaccination". Please justify if this would be better to collect additional data for 120 months (10 years) instead of 90 months (7.5 years). Also, whether the data collection time point of 90 months is based on the protocol?
G.3. This study used a crossover design to evaluate the vaccine efficacy of three groups. The authors should present and discuss the limitations / disadvantages of a cross-over design compared to simple (direct) multi-arm studies.

H. Figures/Tables

H.1. Figure 1. Letter “p” in two boxes at lower left corner cannot be shown completely.
H.2. Please keep “Meningococcal” on a single line.
H.3. For participants assigned to the control group, did they receive “Meningococcal” vaccines in the beginning? If yes, please state this on the plot.

H.4. Figure 2a, 2b. Please remove the background color. Or, include a description for the interpretation (if any) of the faded background color. The authors could consider other simpler approachs to indicate different periods of the study (primary endpoint period, crossover period, etc.).
Use a larger font size for the table of the cumulative incidence of HPV infection.
The x-axis of the incidence plots and the number at risk table should align.
The axis limits of the y-axis of the two plots should be consistent.
H.5. Figure 3a, 3b. The limits of the y-axis of the two smaller plots should be consistent.
H.6. Figure 4a, 4b. Please use a larger font size for the labels and legends.

I. Format
I.1. The number of word counts is 5370, exceeding the suggested word counts of the journal
I.2. Please give space when presenting numeric figures according to common practice. For example, in the Findings section of the Abstract “(95%CI:96.2-99.9%)”, space should be used between “%” and “CI”, and after “CI:”. Also, it is very confusing to use a hyphen “-“ to link the negative confidence limits of a CI, such as “(95%CI:-0.0027--0.0002)”. The negative confidence limits of a CI are commonly separated by a comma and a space “, ”.
I.3. “one-hundred-and-four”, the hyphens are usually not needed.
I.4. Some abbreviations were repeatedly defined in the main text, e.g., VE, and ITT.

(Remarks on code availability)
Data is not available and therefore cannot be reviewed.

Version 1:

Reviewer comments:

Reviewer #1

(Remarks to the Author)
The manuscript is modified in response to my comments, with one minor remaining comment regarding the Vaccine efficacy among adults and young women.
The authors now write:
“Importantly, empiric data are needed on VE among adolescents and young women
after sexual debut, as VE is presumed to be lower due to exposure to HPV.”

I complained about that the sentence does not take into account that vaccine efficacy is from randomised trials known to be high among HPV-negative adolescents and young women. The new sentence has the same problem - it assumes that vaccination is done without HPV testing. VE among untested women is indeed known to be lower when HPV positivity becomes more common. I suggest:
“Importantly, empiric data are needed on VE of one-dose vaccination among HPV-negative adolescents and young women".

(Remarks on code availability)

Reviewer #3

(Remarks to the Author)
Thanks for addressing the previous comments. I have no further comments.

(Remarks on code availability)

**Open Access** This Peer Review File is licensed under a Creative Commons Attribution 4.0 International License, which permits use, sharing, adaptation, distribution and reproduction in any medium or format, as long as you give appropriate credit to the original author(s) and the source, provide a link to the Creative Commons license, and indicate if changes were made.
In cases where reviewers are anonymous, credit should be given to 'Anonymous Referee' and the source.
The images or other third party material in this Peer Review File are included in the article's Creative Commons license, unless indicated otherwise in a credit line to the material. If material is not included in the article's Creative Commons license and your intended use is not permitted by statutory regulation or exceeds the permitted use, you will need to obtain permission directly from the copyright holder.
To view a copy of this license, visit https://creativecommons.org/licenses/by/4.0/

.

**REVIEWER COMMENTS**

Reviewer #1 (Remarks to the Author):

Very clear data on long-term efficacy of one-dose HPV vaccination among adolescent girls. Could have large public health impact on effective strategies for cervical cancer elimination.

We thank the Reviewer for their insightful comments and guidance.

The manuscript is very clear and carefully analysed. Comments:
It is not clear from what source population the participants derive. We are told inclusion and exclusion criteria and enrollment sites. Are they healthy volunteers sourced using advertisements, attending the hospitals for other reasons or enrolled some other way?

This is an important point. In the revised manuscript, we have clarified participant recruitment. We now explicitly state that participants were healthy volunteers recruited through community outreach within the catchment areas of the three Kenya Medical Research Institute (KEMRI) clinical sites. Recruitment did not occur through hospitals or clinical visit-based sampling. This text added to the Methods ("Study design") now reads: "Recruitment was conducted through community outreach activities in surrounding communities, with interested individuals screened for eligibility at study clinics." In the Results, we include that the participants were healthy volunteers.

The impact of the baseline serologies is not clear. As policies are unlikely to use HPV serology at enrollment, a sensitivity analysis ignoring the serology analyses would have been helpful.

We appreciate this suggestion and have added a new sensitivity analysis that excludes all baseline serology information and classifies baseline HPV status using HPV DNA only. This analysis shows VE estimates nearly identical to those in the primary analysis, supporting the robustness of the findings. These results are now included in the Results section and presented in new Supplementary Table SS12.

Most of the estimates in the text are presented with confidence intervals, but not all. It is not useful to state that the incidence is 0.00 if there is no confidence interval or at least a p-value. If the numbers are too small to do the statistics, please just delete these sentences.

We agree and have revised all sections where “0.00” incidence values were previously reported without precision estimates. These have been replaced with descriptive language (e.g., “no cases were observed”), and we removed any numeric incidence reporting where statistical estimation was not possible. We have added CIs consistently.

Finally, I do not understand the sentence “upper age at vaccination at which point VE is presumed to have a small effect on dysplastic lesions and cancer”. The evidence fromRCTs in mid-adult women clearly show high VE in the mITT population (see the Cochrane reviews on this).

Thank you for pointing this out. The sentence has been rewritten for clarity. We now emphasize that randomized trials show high efficacy among HPV-naïve participants, but population-level impact decreases with increasing age at vaccination due to cumulative prior HPV exposure. The revised text removes the ambiguity and now reads: “Importantly, empiric data are needed on VE among adolescents and young women after sexual debut, as VE is presumed to be lower due to exposure to HPV.”

Reviewer #2 (Remarks to the Author):

The MS is very well written, valid methodology even though there were no control as it is not feasible. The study results cover most of the objectives especially on the durability of the single dose at 54 months. However, there some points might consider to be added in the text and especially discuss in the discussion:

1. Data on Table a and b It is noted that the non vaccine types during the cross over vaccination period were much higher than during the enrollment vaccination period in all groups including the control group. Please discuss on this and its effects on the VE and implementation.

Thank you for highlighting this important observation. We have added an explanation in the Results and Discussion noting that the increase in non-vaccine HPV types at the cross over vaccine visit likely reflects aging of the cohort into early adulthood and corresponding increases in exposure risk, including new sexual partnerships. Importantly, the increase is seen across all groups, including participants in the control

group, indicating that this pattern reflects background exposure rather than any vaccine effect. We also clarify that this does not impact VE estimates, which rely only on vaccine-type HPV outcomes.

Text added to the Results: “Reflecting this change, non-vaccine HPV types and gonorrhea prevalence was higher at the cross-over vaccine visit (prevalence of non-vaccine HPV types was 52% and gonorrhea was 2.9% at crossover in the HPV 16/18 mITT cohort) compared to the enrollment vaccine visit (prevalence of non-vaccine HPV types was 37% and gonorrhea was 1.6% at enrollment).”

Text added to the Discussion: “The increase in non-vaccine HPV types and gonorrhea at the cross over vaccine visit compared to the enrollment visit likely reflects aging of the cohort into early adulthood and corresponding increases in exposure risk. Despite the increase in exposure, no difference was seen in VE for vaccine type-specific HPV.”

2. Similar pattern observed for the gonorhoea infections.

Please see our response above.

3. From the Figure 2a and 2b somehow, there is no decline or slightly decine in the cumulative incidence of HPV persistent infections after the delayed HPV vaccination. Please discuss on this and its effects if any as the delayed was about 30-36 motnhs.

Thank you for catching this important point in the Discussion. We have updated the Discussion to clarify that cumulative incidence curves do not decline after vaccination because cumulative incidence is irreversible by definition. Instead, vaccine protection is observed as a reduced slope or plateau in the cumulative-incidence curve. We now explicitly reference the post-crossover incidence rates (rather than cumulative incidence), which demonstrate the expected protective effect.

The Discussion text now reads: “Notably, the incidence rate of persistent HPV infection continued to increase steadily in the control group until crossover vaccination, at which point incidence rate declined to match that of the immediate HPV vaccination group.”

Reviewer #3 (Remarks to the Author):

The authors presented the findings for a clinical trial of the single-dose HPV vaccination. The findings provide evidence-based support for the efficacy of a single-dose schedule. This would help improve access to HPV information and, therefore, help accelerate the time of achieving the goal of cervical cancer elimination, particularly in resource-limited countries.

To help readers understand the content, more details could be provided.

A. Abstract
A.1. The time point at which the crossover was carried out should be included in the Abstract.

We added the timing of crossover vaccination to the Abstract Methods (month 30 or 36).

B.2. Whether the assessment at 54 months was pre-specified in the protocol? If yes, please state this. If not, please justify.

Yes. We now state explicitly in both the Abstract and Methods that the 54-month assessment was pre-specified in the protocol as the endpoint of the follow-up period after crossover vaccination.

Main text
B. Title
B.1. Please include the age range and the targeted vaccinees (female or both sexes) in the title.

We have updated the title to read: "Vaccine efficacy and durability of single-dose human papillomavirus vaccination over 54 months among young women age 15-23 years: a randomized, blinded, cross-over clinical trial"

C. Introduction.
C.1. The introduction could be shortened, given that the current manuscript has exceeded the suggested word counts of the journal.

We have edited the introduction to be shorter.

C.2. Please define "VE" when the term is used in the first

So sorry that we missed this – we have defined the abbreviation when first used.

C.3. The first paragraph of page 5. "In 2023, only 27% of adolescent girls and young women globally were vaccinated against HPV." The authors should report vaccine uptake among low HDI countries. Or, please state clearly if "27%" refers to the uptake among low HDI countries globally.

We revised the text to:

“Globally, in 2023, 27% of adolescent girls, age 9-24 years,  received  HPV vaccination; with coverage in low-and-middle-income countries (LMICs) countries substantially lower.”

Also, what is the age range of “young women” in the statement? Presumably, vaccine uptake decreases as age increases.

We also included that this was 9-14 year-olds.

C.4. The first paragraph of page 5. “… 11.6 million cases of cervical cancer over their lifetimes”. Please state the methodology for getting this number/estimate.

This estimate was from a mathematical modeling study (Bonjour M, Charvat H, Franco EL, et al. Global estimates of expected and preventable cervical cancers among girls born between 2005 and 2014: a birth cohort analysis. The Lancet Public health 2021; 6(7): e510-e21.). In editing the introduction for brevity, we deleted this point.

C.5. The second paragraph of page 5. “Single-dose HPV vaccination has high efficacy over three to five years. ” Please add reference(s) for this statement, and state the condition for the findings. If the single-dose vaccination schedule has been shown/reported for having a high efficacy over 3-5 years, please include the added value for the current study that evaluated the efficacy at 54 months, i.e., 4.5 years, which is within the range of 3-5 years.

Thank you for helping us clarify this point. References to prior RCTs and observational studies have been added.[15-18, 23] We emphasize that our trial provides the first randomized, blinded, long-term durability results extending beyond prior published follow-up periods.

D. Study objective.
D.1. What is the maximum age of the study? It is unclear to state “15 years an older”.

We agree and have modified that to read 15-23 years.

D.2. State the rationale for evaluating the efficacy at 54 months, i.e., 4.5 years, instead of a time point of an integer in years, in addition to referring readers to the protocol. Also, the author mentioned that this was “based on sustained antibody levels observed over 16 years”. What is the connection between 54 months and 16 years?

We explain that 54 months corresponds to the predefined blinded follow-up period after crossover vaccination. The mention of 16-year antibody durability refers to broader scientific evidence supporting long-term durability but is independent of the 54-month endpoint. This has now been clarified.

E. Methods.

E.1. Please refer the readers to the flow chart when describing the vaccination schedules. The study design is unclear from the text only.

We now explicitly reference Figure 1 (CONSORT) during description of the vaccination schedules.

E.2. Study design. The paragraph “In GAVI-funded countries, …” is more related to the rationale of the study, rather than the methods. Consider moving the paragraph to a better position.

We agree that this point is best made in the introduction, which now covers it.

E.3. Cite references for the assessment of durability “If the vaccine is not durable (i.e., the vaccine effect wanes), we expect the coefficient for time since vaccination to be positive …” (page 12).

The coefficient is a property of the equation, and we were not able to identify a specific reference that would discuss the interpretation of this parameter. However, this general approach is covered by the Follmann reference (Follmann D, Fintzi J, Fay MP, et al. A Deferred-Vaccination Design to Assess Durability of COVID-19 Vaccine Effect After the Placebo Group Is Vaccinated. Ann Intern Med 2021; 174(8): 1118-25.).

E.4. For the assessment of durability, the modified intention-to-treat (mITT) cohort/approach was considered. For the assessment of vaccine effectiveness, the intention-to-treat (ITT) cohort/approach was considered. The authors should explain the difference.

We added text defining mITT (excludes HPV infection at baseline) and ITT (includes all participants regardless of baseline status) and explain why each was used.

The text reads: “We used both a modified intention-to-treat (mITT) and an intention-to-treat (ITT) cohort. The mITT cohort excluded participants with evidence of baseline HPV infection with the outcome HPV types and therefore estimates vaccine efficacy among those HPV-naïve at vaccination. The ITT cohort included all randomized participants

regardless of baseline infection status and reflects vaccine effectiveness under real-world conditions where pre-vaccination HPV testing is not performed."

F. Results.
F.1. Second paragraph, page 15. "The incidence of persistent non-vaccine HPV types was comparable …" Please indicate the p-value of the comparison of the incidence between groups.

A priori, we were not powered to do a test of equality as a predefined analysis. We calculated the confidence interval for the difference in incidence rates and added this to Table S5.

F.2. Please indicate the findings of "negative coefficients of time since vaccination" in the Results section.

Thank you for highlighting this – we have added the number from the Figures to the text.

G. Discussion
G.1. It would be interesting to discuss the rationale of the requirement of receiving three doses when HPV vaccines (both bivalent and quadrivalent by different manufacturers) were first available in the market.

The Discussion now includes that this was based on Hep B subunit vaccine and the prime boost approach. The text now reads: "While the three-dose schedule was based on the hepatitis B subunit vaccines, a single-dose of the HPV vaccine has high and durable efficacy."

G.2. When stating the limitations, the authors mentioned that "observational data for single-dose HPV vaccination support efficacy over a decade … We are collecting additional data in this cohort to 90 months post-vaccination". Please justify if this would be better to collect additional data for 120 months (10 years) instead of 90 months (7.5 years). Also, whether the data collection time point of 90 months is based on the protocol?

We note that 90 months is the protocol-specified funded follow-up period. This has now been stated clearly. We agree that extension to 120 months would be ideal but requires additional resources.

G.3. This study used a crossover design to evaluate the vaccine efficacy of three

groups. The authors should present and discuss the limitations / disadvantages of a cross-over design compared to simple (direct) multi-arm studies.

We agree that a crossover design has inherent limitations, including the loss of a contemporaneous unvaccinated control group after crossover and reliance on modeled durability estimates rather than direct comparison with an ongoing placebo arm. We note these limitations explicitly and explain that the design was ethically appropriate once efficacy was established and helped minimize attrition while still allowing rigorous assessment of long-term vaccine performance.

The text now reads: "A crossover design inherently limits long-term comparisons because a contemporaneous unvaccinated control group is no longer available after crossover, requiring durability to be assessed using modeled estimates rather than direct placebo comparisons. Nonetheless, this design was ethically appropriate once efficacy was demonstrated and minimized attrition while still enabling robust evaluation of long-term vaccine performance."

H. Figures/Tables
H.1. Figure 1. Letter "p" in two boxes at lower left corner cannot be shown completely.
H.2. Please keep "Meningococcal" on a single line.
H.3. For participants assigned to the control group, did they receive "Meningococcal" vaccines in the beginning? If yes, please state this on the plot.

H.4. Figure 2a, 2b. Please remove the background color. Or, include a description for the interpretation (if any) of the faded background color. The authors could consider other simpler approachs to indicate different periods of the study (primary endpoint period, crossover period, etc.).
Use a larger font size for the table of the cumulative incidence of HPV infection.
The x-axis of the incidence plots and the number at risk table should align.
The axis limits of the y-axis of the two plots should be consistent.
H.5. Figure 3a, 3b. The limits of the y-axis of the two smaller plots should be consistent.
H.6. Figure 4a, 4b. Please use a larger font size for the labels and legends.

Thank you for these suggestions which have improved the figures and their readability. All figure formatting changes (axis alignment, removal of background shading, consistent y-axis limits, improved font sizes, correcting "Meningococcal," and clarifying that control participants received meningococcal vaccination at baseline) have been completed.

I. Format
I.1. The number of word counts is 5370, exceeding the suggested word counts of the

journal
I.2. Please give space when presenting numeric figures according to common practice. For example, in the Findings section of the Abstract “(95%CI:96.2-99.9%)”, space should be used between “%” and “CI”, and after “CI:”. Also, it is very confusing to use a hyphen “-“ to link the negative confidence limits of a CI, such as “(95%CI:-0.0027--0.0002)”. The negative confidence limits of a CI are commonly separated by a comma and a space “, ”.
I.3. “one-hundred-and-four”, the hyphens are usually not needed.
I.4. Some abbreviations were repeatedly defined in the main text, e.g., VE, and ITT.

We have edited and tightened the text, standardized CI formatting, removed unnecessary hyphens, and eliminated repeated abbreviation definitions.

Reviewer #3 (Remarks on code availability):

Data is not available and therefore cannot be reviewed.

Data cannot be shared publicly because this study was conducted with approval from the Kenya Medical Research Institute (KEMRI) Scientific and Ethics Review Unit (SERU), which requires that data from studies (including de-identified data) are released only after SERU has provided written approval for additional analyses. A complete de-identified dataset sufficient to reproduce the study findings will be made available upon written request after approval from SERU. To request these data, please contact the KEN SHE Scientific Committee at lnakatsuka@partners.org.

We are grateful for this opportunity to submit a revised manuscript. Please let me know if there are additional questions.

Warm regards,

Ruanne Barnabas on behalf of the KEN SHE Team

**REVIEWER COMMENTS**

Reviewer #1 (Remarks to the Author):

The manuscript is modified in response to my comments, with one minor remaining comment regarding the Vaccine efficacy among adults and young women.

The manuscript has benefit immensely from the Reviewers' comments – thank you!

The authors now write:

“Importantly, empiric data are needed on VE among adolescents and young women

after sexual debut, as VE is presumed to be lower due to exposure to HPV.”

I complained about that the sentence does not take into account that vaccine efficacy is from randomised trials known to be high among HPV-negative adolescents and young women. The new sentence has the same problem - it assumes that vaccination is done without HPV testing. VE among untested women is indeed known to be lower when HPV positivity becomes more common. I suggest:

“Importantly, empiric data are needed on VE of one-dose vaccination among HPV-negative adolescents and young women".

We understand that this is an important point and the Reviewer is correct that HPV VE is demonstrated among HPV naïve persons. We have modified the manuscript to read: “Importantly, empiric data are needed on VE of one-dose vaccination among HPV-negative adolescents and young women.”
